# Impacts of *CD36* Variants on Plasma Lipid Levels and the Risk of Early-Onset Coronary Artery Disease: A Systematic Review and Meta-Analysis

**DOI:** 10.1155/cdr/8098173

**Published:** 2025-02-25

**Authors:** Zhi Luo, Lingwei Lv

**Affiliations:** ^1^Department of Cardiology, Suining Central Hospital, Suining, Sichuan, China; ^2^Department of Orthopedics, The Fifth Affiliated Hospital of Guangzhou Medical University, Guangzhou, Guangdong, China

**Keywords:** CD36, dyslipidemia, early-onset coronary artery disease, genetic polymorphism

## Abstract

**Background:** Recent studies have indicated that cluster of differentiation 36 (CD36) is closely linked to dyslipidemia and early-onset coronary artery disease (EOCAD). This study is aimed at investigating the impacts of *CD36* gene variants on lipid profiles and EOCAD risk.

**Methods:** PubMed, Cochrane Library, Central, CINAHL, and ClinicalTrials.gov were searched until June 15, 2024.

**Results:** In total, 25 studies (11,494 individuals) were included for the analysis. The A allele carriers of the rs1761667 variant had higher high-density lipoprotein cholesterol (HDL-C) levels and higher EOCAD risk than noncarriers. In contrast, the G allele carriers of the rs1049673 and rs3211956 variants had lower low-density lipoprotein cholesterol (LDL-C) levels and lower EOCAD risk than noncarriers. Subgroup analysis indicated that the antiatherosclerotic impact and reduced EOCAD risk were primarily observed in Chinese with rs1049673 and rs3211956.

**Conclusions:** The rs1761667, rs1049673, and rs3211956 variants of the *CD36* gene have significant impacts on lipid levels and may serve as genetic markers for the risk of EOCAD primarily in Chinese. The impacts of *CD36* variants on EOCAD risk are mediated, at least partly, by dyslipidemia. Genetic screening of *CD36* gene variants may be helpful for early intervention or prevention of EOCAD in individuals with high risk factors.

## 1. Introduction

The cluster of differentiation 36 (CD36) is a transmembrane glycoprotein of the class B scavenger receptor family [1], also known as scavenger receptor class B type I, scavenger receptor class B member 3, fatty acid translocase, glycoprotein IIIb, platelet membrane glycoprotein IV, thrombospondin receptor, platelet collagen receptor, and posterior aspect of segment IV [1], which is predominantly expressed in cardiac myocytes, microvascular endothelial cells, hepatocytes, adipocytes, macrophages, and platelets [2, 3].

CD36 is a receptor of natural lipoproteins and recognizes and uptakes a wide variety of lipid ligands, including long-chain fatty acid, low-density lipoprotein, very-low-density lipoprotein, high-density lipoprotein, and oxidized low-density lipoprotein (OxLDL) [4, 5]. However, the function of CD36 in several hepatocyte experiments was controversial. For instance, Truong et al. [6] found that overexpression of CD36 in HepG2 cells increased cholesterol efflux by 92%, whereas knockout of CD36 in normal hepatocytes did not influence the cholesterol efflux rate [6]. In contrast, Yue et al. [7] reported that CD36-null hepatocytes have higher cholesterol efflux rates than wild-type hepatocytes. Moreover, Brundert et al. [8] reported that hepatic CD36 increased the selective uptake of high-density lipoprotein cholesterol (HDL-C) by hepatocytes. In sharp contrast to these cell experiments, a series of mouse model experiments showed more consistent results. For instance, Febbraio et al. [9] found that loss-of-function mutation in mice *CD36* gene significantly increased fasting cholesterol and triglyceride (TG) levels. Consistently, knockout of the *CD36* gene in mice increased the synthesis of lipoprotein particles smaller than CM [10]. Conversely [9, 10], overexpression of the *CD36* gene in mice decreased cholesterol and TG levels [11]. It indicated that CD36 protein levels or function might be critical in lipid metabolism homeostasis. Since the genetic variants of the *CD36* gene may affect CD36 protein expression levels [12–14], in addition to CD36 protein levels being closely linked to lipid metabolism [10, 11], it indicated that *CD36* genetic variants might affect lipid levels by regulating CD36 protein levels or function [15, 16].

Developing a genetically targeted *CD36* null mouse line has played a critical role in demonstrating in vivo relevance to atherosclerosis disorder [9]. For instance, macrophages from *CD36* knockout mice bind and internalize substantially less OxLDL than those from wild-type mice and consequently do not effectively form foam cells [17]. In contrast, macrophages from *CD36* knockout mice had decreased proinflammatory characteristics and less migration to a proinflammatory stimulus compared to apolipoprotein E (*APOE*) knockout or scavenger receptor A (*SRA*) knockout mice [18]. Additionally, breeding *CD36* knockout mice into several different atherosclerosis-prone mouse strains, including *APOE* knockout and low-density lipoprotein receptor (*LDLR*) knockout, substantially reduced the amount of plaque formed in these mouse models [19–21]. The degree of atheroprotection differs in these animal models, but all showed either smaller lesions or less complex lesions [19–21]. Interestingly, the impacts of *CD36* deficiency on atherosclerosis in mice were sex-specific, for example, knockout of *CD36* in male mice increased carbohydrate utilization and decreased energy expenditure, while knockout of *CD36* in female mice reduced atherosclerosis [22].

Recently, Touré et al. [23, 24] conducted two promising studies to investigate the correlation between plasma soluble CD36 (sCD36) levels and dyslipidemia. Their results indicated that plasma sCD36 levels were negatively correlated with plasma HDL-C and TG levels in healthy subjects and positively correlated with total cholesterol (TC) levels in patients with Type 2 diabetes [23]. In contrast, increased plasma levels of sCD36 would be associated with lower TC levels, higher low-density lipoprotein cholesterol (LDL-C) levels in healthy subjects, and higher TG levels in obese diabetic patients [24]. sCD36 is primarily shed from circulating cells such as platelets, monocytes/macrophages, or adipocytes [25]; the function of sCD36 is still unknown, may just be a marker of CD36 on blood cells or another source. Therefore, further studies are needed to clarify its specific function.

CD36 is involved in multiple pathophysiological processes. For instance, Asch et al. [26] conducted a site-directed mutagenesis study by altering the threonine phosphorylation site to alanine and found that CD36 was expressed in a dephosphorylated state, indicating that CD36 may affect signal transduction. Qi et al. [27] performed a set of elegant experiments in the FeCl3-injured mesenteric arteriole thrombosis mouse model. They found that CD36 activated a series of signaling pathways and thus enhanced platelet activation and myocardial infarction expansion, indicating that CD36 may affect thrombosis in vivo. In addition, transfection of *CD36* gene into human Bowes melanoma cells was found to increase the capacity to ingest apoptotic neutrophils, lymphocytes, and fibroblasts [28], indicating that CD36 may be related to phagocytosis and apoptosis.

The CD36 protein is encoded by the *CD36* gene [1, 12], which is located on the long arm of chromosome 7 (7q21.11), including 15 exons [1]. Of these, Exons 1, 2, 15, and the first 89 nucleotides of Exon 3 are noncoding. Moreover, Exons 3 and 14 encode the N-terminal and C-terminal domains of the *CD36* mRNA, respectively [29, 30]. rs1761667, also known as −31118 G>A, is located in 5⁣′ flanking Exon 1A, formed by transversion from guanine (G) to adenine (A). rs1049673, also known as 30294 C>G, is located in the 3⁣′-untranslated regions (3⁣′-UTR) of Exon 15, formed by transversion from cytosine (C) to G. rs3211956 is located in the 3⁣′-UTR of Intron 14, formed by transversion from thymine (T) to G.

Early-onset coronary artery disease (EOCAD) is defined as the initial manifestation of coronary artery disease (CAD) in males under the age of 55 years and females under the age of 65 years [31]. Early-onset myocardial infarction, a life-threatening consequence of EOCAD, is a leading cause of death and disability in the young population. Previous studies found that the A allele of rs1761667 [32], the C allele of rs1049673 [33], and the T allele of rs3211956 [33] had a higher frequency distribution in EOCAD patients compared to those healthy subjects. It indicated that the A allele of rs1761667 might increase, while the G allele of rs1049673 and rs3211956 might decrease the risk of EOCAD. Since dyslipidemia is one of the most critical risk factors for CAD and accounts for at least 50% of population-attributable risk [34], it is tempting to speculate that the increased EOCAD risk associated with *CD36* variants [32, 33] may stem from atherogenic dyslipidemia. To verify this hypothesis, we conducted this study to investigate the impacts of *CD36* variants on lipid profiles and EOCAD risk.

Here, a total of 25 studies [23, 24, 32, 33, 35–55] were enrolled in this meta-analysis to investigate the impacts of *CD36* variants on plasma lipid levels and the risk of EOCAD.

## 2. Methods

The current meta-analysis follows the Preferred Reporting Items for Systematic Reviews and Meta-Analyses (PRISMA) 2020 Checklist (http://www.prisma-statement.org/PRISMAStatement/) [56]. The registration information including the registration number is not available.

### 2.1. Literature Search

A comprehensive literature search was performed from January 5, 2021, to June 15, 2024, using PubMed, Cochrane Library, Central, CINAHL, and ClinicalTrials.gov. The following keywords were used in the search: [“CD36,” “fatty acid translocase,” “FAT,” “SR-BI,” “SRB3,” “GPIIIB,” “GPIV,” “TSP,” “PAS IV,” “rs1761667,” “rs1049673,” “rs3211956”] AND [“polymorphism,” “mutation,” “variation,” “mutant,” “variant,” “SNP”] OR [“single nucleotide polymorphism”] AND [“lipids,” “circulating lipids,” “blood lipids,” “plasma lipids,” “serum lipids,” “lipid profile”] OR [“triglycerides,” “total cholesterol,” “low-density lipoprotein cholesterol,” “high-density lipoprotein cholesterol,” “TG,” “TC,” “LDL-C,” “HDL-C”] AND/OR [“early-onset coronary artery disease,” “premature coronary artery disease,” “EOCAD,” “PCAD”]. Additionally, the reference lists of all eligible studies were manually retrieved to search for additional literature.

### 2.2. Inclusion Criteria

The inclusion criteria for the impacts of the *CD36* variants on EOCAD risk include (1) studies using a population-based case–control design; (2) EOCAD angiographically defined; and (3) genotype frequencies of rs1761667, rs1049673, and rs3211956 in EOCAD and control groups available. The inclusion criteria for the impacts of the *CD36* variants on lipid profiles include the following: (1) Studies investigated the association of rs1761667, rs1049673, and rs3211956 with plasma lipid levels. (2) Studies at least provided three of four parameters in lipid profiles (TG, TC, LDL-C, and HDL-C). (3) Studies provided genotype frequencies of rs1761667, rs1049673, and rs3211956. (4) Studies provided mean lipid levels with standard deviation (SD) or standard error (SE) by the genotype of rs1761667, rs1049673, and rs3211956. (5) Interventional studies provided preintervention data. (6) The language of eligible studies was restricted to English or Chinese. The exclusion criteria for this study include (1) studies that did not relate to rs1761667, rs1049673, and rs3211956; (2) studies that did not provide the counts of genotype or allele; (3) pedigree or overlapping studies; and (4) abstract, comment, letter, case report, review, and animal studies.

### 2.3. Data Extraction

Lingwei Lv extracted the data by using standardized data extraction tables. Main data points included study details (first author's name, year, country, ethnicity, gender, genotype counts, genotyping methods, study design, and study period), total counts of patients, and mean lipid levels with SD or SE by genotype.

### 2.4. Data Analysis

TG, TC, LDL-C, and HDL-C units were converted into millimoles/liter. All extracted data were expressed as mean ± SD. Odds ratio (OR) with 95% confidence interval (CI) was used to evaluate the associations of rs1761667, rs1049673, and rs3211956 with the risk of EOCAD. Standardized mean difference with 95% CI was used to evaluate the differences in lipid levels between the genotypes of rs1761667, rs1049673, and rs3211956. The pooled OR was performed for allelic model [(A vs. G) for rs1761667, (G vs. C) for rs1049673, and (G vs. T) for rs3211956], additive model [(AA vs. GG) for rs1761667, (GG vs. CC) for rs1049673, and (GG vs. TT) for rs3211956], dominant model [(GA + AA) vs. GG for rs1761667, (CG + GG) vs. CC for rs1049673, and (TG + GG) vs. TT for rs3211956], and recessive model [(GG + GA) vs. AA for rs1761667, (CG + CC) vs. GG for rs1049673, and (TG + TT) vs. GG for rs3211956]. Since most of the included studies presented lipid data in a dominant model [(GA + AA) vs. GG for rs1761667, (CG + GG) vs. CC for rs1049673, and (TG + GG) vs. TT for rs3211956], a dominant model was adopted to ensure adequate statistical power. All statistical tests were conducted with the Cochrane Collaboration meta-analysis software (Review Manager 5.4). *p* < 0.05 was recognized as statistically significant.

### 2.5. Subgroup Analysis

Subgroup analysis was performed by ethnicity, gender, and health status. The ethnicity was divided into Caucasian and Chinese individuals. Healthy status was divided into CAD and the general population. In some studies, subjects were divided into multiple subpopulations (e.g., subjects with different types of disease, subjects originating from different races, and case and control subjects). Each subpopulation was regarded as an independent comparison in this study.

### 2.6. Evaluation of Heterogeneity

Heterogeneity was tested by the *I*^2^ statistic and Cochran's *χ*^2^-based Q statistic. If heterogeneity was significant (*I*^2^ > 50%, p ≤ 0.05), a random-effects model (DerSimonian–Laird method) was used to calculate the results [57]. Otherwise, a fixed-effects model (Mantel–Haenszel method) was adopted (*I*^2^ < 50%, p > 0.05). In addition, the Galbraith plot was employed to detect the potential sources of heterogeneity. To completely eliminate the impact of heterogeneity on results, all results were recalculated after excluding studies with heterogeneity.

### 2.7. Sensitivity Analysis

Sensitivity analysis was performed by omitting risky comparisons one by one. If the recalculated results did not change substantially after omitting each risky comparison, it indicated the robustness of the analysis results.

### 2.8. Publication Bias Test

The Begg funnel plot and Egger linear test evaluated the probability of publication bias among the included studies [58].

### 2.9. The Primary and Secondary Results of This Study

#### 2.9.1. Primary Results

1. The impacts of *CD36* variants (i.e., rs1761667, rs1049673, and rs3211956) on TG, TC, LDL-C, and HDL-C in an integrated population (i.e., Caucasian and Chinese individuals)
2. The impact of *CD36* variants (i.e., rs1761667, rs1049673, and rs3211956) on PCAD risk in the genetic models of allelic, additive, dominant, and recessive

#### 2.9.2. Secondary Results

1. The impacts of *CD36* variants (i.e., rs1761667, rs1049673, and rs3211956) on TG, TC, LDL-C, and HDL-C in specific populations, including Caucasian individuals, Chinese individuals, male individuals, and CAD patients
2. The impact of rs1800795 on PCAD risk in Caucasian or Chinese individuals under the genetic models of allelic, additive, dominant, and recessive

## 3. Results

### 3.1. Study Selection

**Figure 1:**
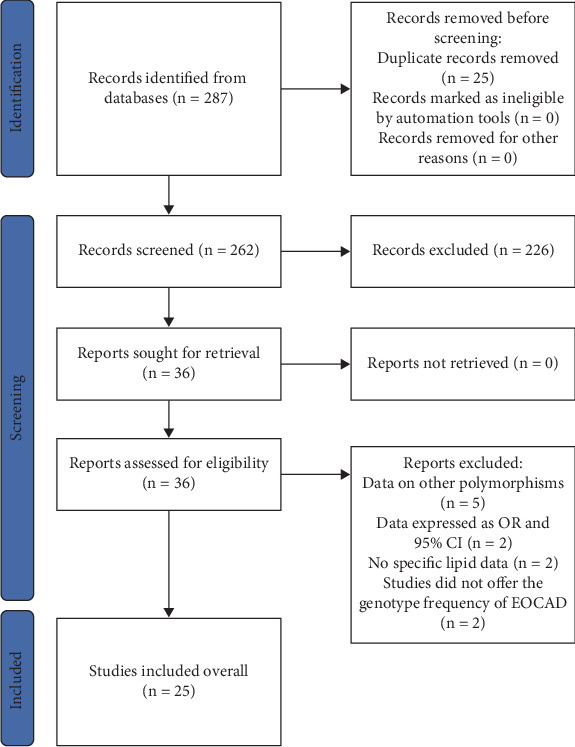
Flow diagram of the studies' selection process.

The details of the study selection are summarized in Figure 1.

### 3.2. Characteristics of the Included Studies

The present study included 25 studies (11,494 individuals). Characteristics of studies for CD36 variants are presented in Table S1. Characteristics of studies for rs1761667 are presented in Table S2. Characteristics of studies for rs1049673 are presented in Table S3. Characteristics of studies for rs3211956 are presented in Table S4. Plasma lipid levels by rs1761667 are presented in Table S5. Plasma lipid levels by rs1049673 are presented in Table S6. Plasma lipid levels by rs3211956 are presented in Table S7.

The forest plot for rs1761667 with EOCAD is presented in Figure S1. The forest plot for rs3211956 with EOCAD is presented in Figure S2. Sensitivity analysis for rs1049673 with lipid levels is presented in Figure S3. Sensitivity analysis for rs3211956 with lipid levels is presented in Figure S4. Sensitivity analysis for rs3211956 with EOCAD is presented in Figure S5. Sensitivity analysis for rs1761667 with EOCAD is presented in Figure S6. Sensitivity analysis for rs1761667 with lipid levels is presented in Figure S7. Begg's funnel plot for rs1761667 with lipid levels is presented in Figure S8. Begg's funnel plot for rs1049673 with lipid levels is presented in Figure S9. Begg's funnel plot for rs3211956 with lipid levels is presented in Figure S10. Begg's funnel plot for rs1761667 with EOCAD is presented in Figure S11. Begg's funnel plot for rs1049673 with EOCAD is presented in Figure S12. Begg's funnel plot for rs3211956 with EOCAD is presented in Figure S13.

### 3.3. The Incidence of *CD36* Mutations

The incidence of rs1761667 A allele, rs1049673 G allele, and rs3211956 G allele in the CAD group was 44.0% (range from 39.1% to 48.9%), 46.4% (range from 41.6% to 51.2%), and 22.6% (range from 18.0% to 27.2%), respectively (Tables S2–S4). The incidence of rs1761667 A allele, rs1049673 G allele, and rs3211956 G allele in the control group was 41.5% (range from 36.8% to 46.2%), 52.5% (range from 44.3% to 60.7%), and 25.8% (range from 18.3% to 33.3%), respectively (Tables S2–S4). In addition, the incidence of rs1761667 A allele and rs1049673 G allele in Caucasian individuals was 48.4% (range from 36.8% to 60.0%) and 51.95% (range from 49.1% to 54.8%), respectively (Table S5 and Table S6). In contrast, the incidence of rs1049673 G allele and rs3211956 G allele in Chinese individuals was 51.1% (range from 47.0% to 55.2%) and 25.3% (range from 21.0% to 29.6%), respectively (Table S6 and Table S7).

### 3.4. Impact of rs1761667 on Lipid Profile

**Figure 2:**
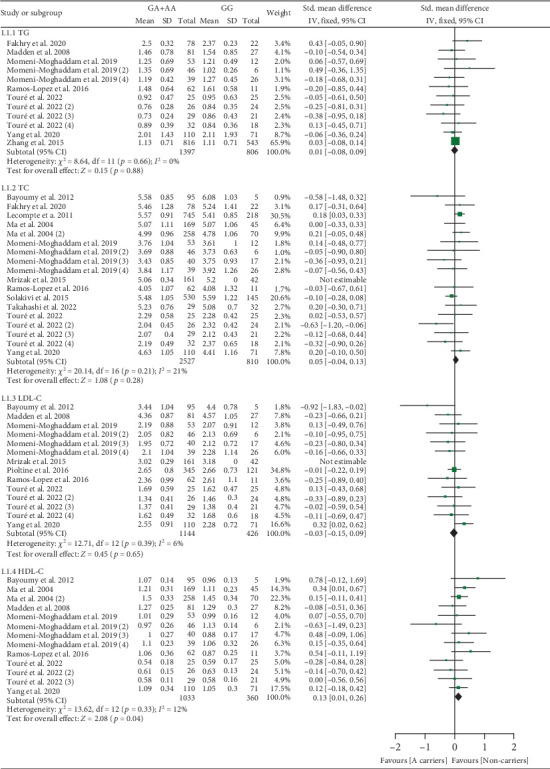
Forest plot of the meta-analysis between *CD36* rs1761667 variant and plasma lipid levels.

All results below were data with heterogeneity excluded. The rs1761667 A allele increased HDL-C levels (Table S8 and Figure 2). Subgroup analysis indicated that the impact of rs1761667 on HDL-C levels was significant in Caucasian individuals (Table S8).

### 3.5. Impact of rs1049673 on Lipid Profile

**Figure 3:**
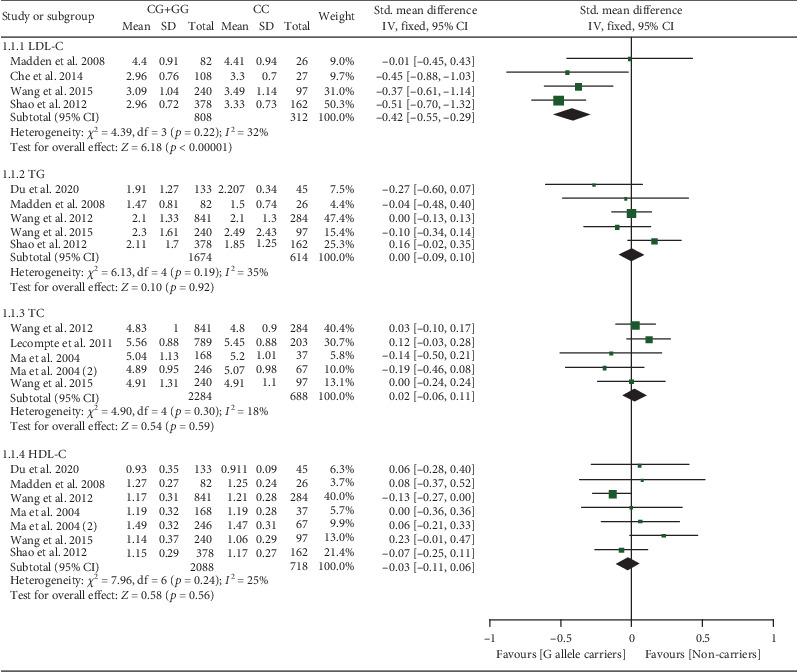
Forest plot of the meta-analysis between *CD36* rs1049673 variant and plasma lipid levels.

The rs1049673 G allele decreased LDL-C levels (Table S9 and Figure 3). Subgroup analysis indicated that the impact of rs1049673 on LDL-C levels was significant in Chinese individuals (Table S9).

### 3.6. Impact of rs3211956 on Lipid Profile

**Figure 4:**
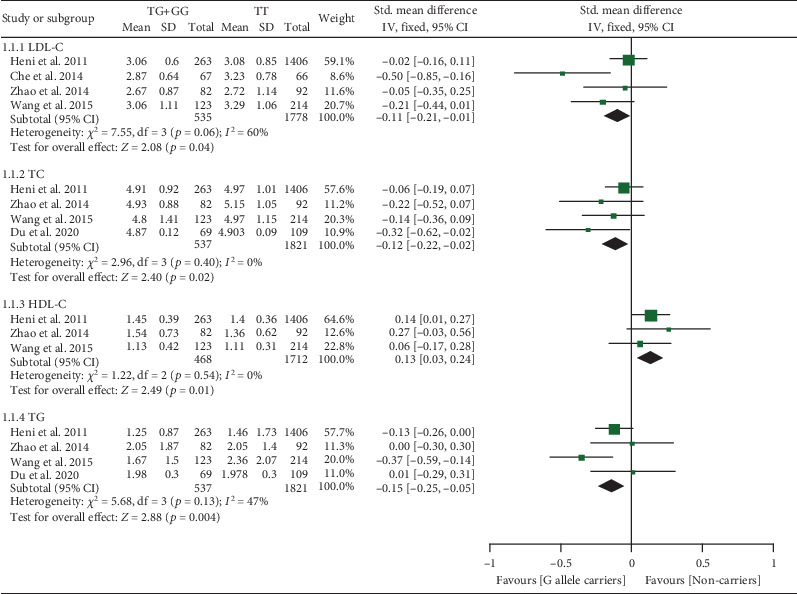
Forest plot of the meta-analysis between *CD36* rs3211956 variant and plasma lipid levels.

The rs3211956 G allele decreased TG, TC, and LDL-C and increased HDL-C levels (Table S10 and Figure 4). Subgroup analysis indicated that the impacts of rs3211956 on lipid profiles were significant in Chinese individuals (Table S10).

### 3.7. Impact of rs1761667 on EOCAD Risk

The A allele of rs1761667 increased EOCAD risk under dominant and recessive models (Table S11 and Figure S1). Subgroup analysis indicated that the impact of rs1761667 on EOCAD risk was significant in Caucasian individuals (Table S11).

### 3.8. Impact of rs1049673 on EOCAD Risk

**Figure 5:**
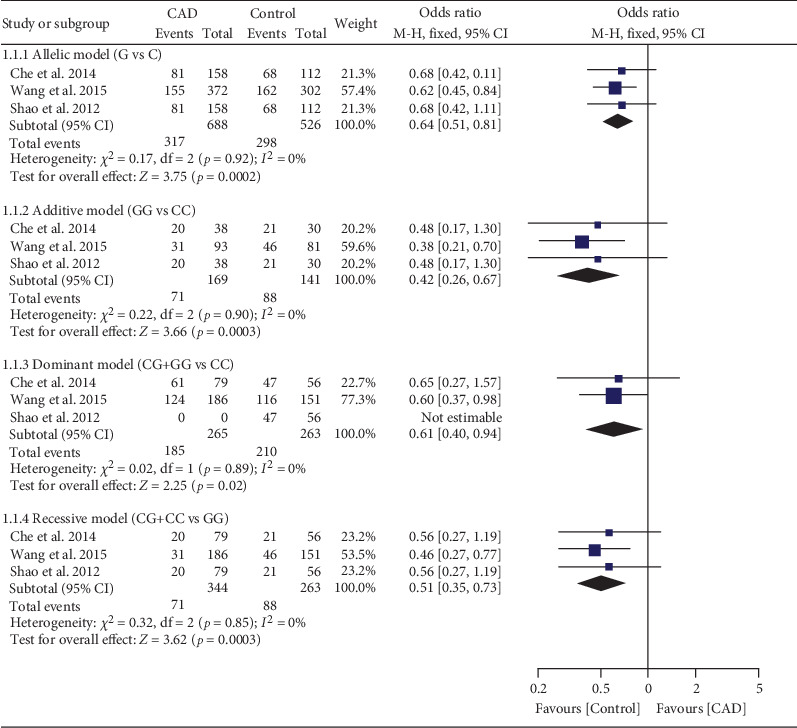
Forest plot of the meta-analysis between *CD36* rs1049673 variant and early-onset coronary artery disease risk.

The G allele of rs1049673 decreased EOCAD risk under all genetic models (Table S12 and Figure 5). Subgroup analysis indicated that the impact of rs1049673 on EOCAD risk was significant in Chinese individuals (Table S12).

### 3.9. Impact of rs3211956 on EOCAD Risk

The G allele of rs3211956 reduced EOCAD risk under allelic and dominant models (Table S13 and Figure S2). Subgroup analysis indicated that the impact of rs3211956 on EOCAD risk was significant in Chinese individuals (Table S13).

### 3.10. Evaluation of Heterogeneity

Significant heterogeneity was detected among *CD36* variants, lipid levels, and EOCAD risk (Tables S8–S10, Table S12, and Table S13). Notably, the recalculated results changed substantially after excluding the studies with heterogeneity (see Tables S8–S10, Table S12, and Table S13 for more details).

### 3.11. Sensitivity Analysis

Sensitivity analysis indicated that one comparison [48] might affect the impacts of rs1049673 on TG and LDL-C levels (Figure S3), one comparison [46] might affect the impact of rs1049673 on HDL-C levels (Figure S3), one comparison [49] might affect the impacts of rs3211956 on TC and LDL-C levels (Figure S4), one comparison [47] might affect the impact of rs3211956 on EOCAD risk (Figure S5), and one comparison [52] might affect the impact of rs1761667 on EOCAD risk (Figure S6). Moreover, Lecompte et al. [37], Pioltine et al. [42], and Zhang et al. [39] analyzed what might affect the impacts of rs1761667 on TC, LDL-C, and HDL-C levels (Figure S7). Interestingly, the recalculated results did not change substantially after omitting these comparisons. It indicated the robustness of the analysis results.

### 3.12. Publication Bias Test

The Begg funnel plot was used to evaluate publication bias among the included studies. And no publication bias was detected in this meta-analysis, which was confirmed by the Egger linear regression test (see Figures S8–S13 for more details).

## 4. Discussion

The present study indicated that *CD36* variants had considerable impacts on blood lipid levels and EOCAD risk.

CD36 antigen deficiency is relatively frequent in eastern populations [59], which generates different impacts on specific populations. For instance, Nozaki and colleagues [60] analyzed the monocyte-derived macrophages from normal and CD36-deficient Japanese populations; they found that cholesteryl ester mass accumulation was reduced by approximately 40% in the macrophages from CD36-deficient subjects than those from normal controls after incubation with OxLDL for 24 h. In contrast, Lo et al. [61] used flow cytometric methods to study the CD36 deficiency in 640 regular volunteer platelet apheresis donors from the Taipei Blood Center; they found that Taiwanese with null or low platelet CD36 expression were more likely to have a lipemic deferral record than control subjects with normal platelet CD36 expression (*X*(2) = 27.36, OR = 2.6, 95% CI: 1.8–3.8, *p* < 0.0001). In addition, Masuda et al. [62] and Xu et al. [63] analyzed the expression levels of CD36 on platelets and monocytes in the Japanese and Chinese populations, respectively. They found that CD36 expression in platelets and monocytes from Type II-deficient (lacking CD36 expression on platelets only) subjects with CD36 gene variants was lower than that in platelets and monocytes from Type II-deficient subjects without gene variants. In a Thai-based study, Phuangtham et al. [64] analyzed the expression and deficiency of CD36 on platelets and monocytes by flow cytometry; they found that CD36 Type I deficiency (lacking CD36 expression on platelets and monocytes) indicated the potential for immune-mediated platelet disorders, and sCD36 could be used to identify individuals at risk.

Variants of *CD36* remodeled lipid profiles primarily through the following two pathways: (1) inhibiting the expression or function of CD36 protein. CD36 is closely related to lipid metabolism homeostasis [65–67]. Increasing CD36 expression or function ameliorates lipid metabolism [6, 11], while knocking out CD36 [10] or loss of function in CD36 [9] deteriorates lipid metabolism. Therefore, rs1761667 may result in dyslipidemia by inhibiting the expression or function of the CD36 protein [15, 16]. However, Truong et al. [6] and Yue et al. [7] reported that the knockout of CD36 did not influence cholesterol efflux rates in normal hepatocytes. It indicates that the protein function of CD36 is ambiguous and needs to be confirmed by more functional experiments; (2) the second is reduction of plasma levels of sCD36. Touré et al. [23] reported that the rs1761667 A allele decreased plasma sCD36 levels, and sCD36 levels were negatively associated with HDL-C levels [23]; it is tempting to speculate that the A allele of rs1761667 is associated with higher levels of HDL-C. Intriguingly, this hypothesis was verified in Table S1. Also, rs1049673 and rs3211956 may affect lipid levels through the mediation of sCD36 [23, 24]. Further studies are needed to verify this speculation.

The G allele of rs1049673 largely lowered LDL-C levels (Table S9) and EOCAD risk (Table S12). Since LDL-C plays a central role in CAD pathogenesis [34], it indicated that the beneficial impact of rs1049673 on EOCAD (Table S12) was mediated, at least partly, by the decreased LDL-C levels (Table S9). Consistently, the rs3211956 G allele ameliorated lipid profiles (Table S10) and reduced EOCAD risk (Table S13), indicating that the protective impact of rs3211956 on EOCAD (Table S13) was mediated by the ameliorated lipid metabolism (Table S10).

Additionally, the rs1761667 A allele elevated HDL-C levels (Table S8) and increased EOCAD risk (Table S11). According to Chiesa et al. [68] and Favari et al. [69] studies, carriers of the apolipoprotein A-I(Milano) (A-I(M)) variant presented with severe reductions of plasma HDL-C levels but had less risk of suffering EOCAD, suggesting that the function of the HDL-C particles was more important than the level. Likewise, most of the drugs inhibiting CETP increased HDL-C substantially but had no cardiovascular benefit [70]. This evidence [68–70] indicated that HDL-C was not always protective and even led to an increased risk of cardiovascular events and deaths. Therefore, the association between rs1761667 A allele and the increased risk of EOCAD (Table S11) was possibly mediated by elevated HDL-C levels (Table S8).

According to the newest management strategies for EOCAD [71], tobacco use, elevated blood pressure/hypertension, family history of premature atherosclerotic cardiovascular disease, primary severe hypercholesterolemia such as familial hypercholesterolemia, diabetes with diabetes-specific risk-enhancing factors, or the presence of multiple other risk-enhancing factors, including in females, a history of pre-eclampsia or menopause under age 40 were recognized as the primary risk factors for EOCAD. Since rs1761667 was linked with hypertension [32], diabetes [72], and obesity [73], but not TC, LDL-C, or HDL-C levels (Table S8), it indicated that the impact of rs1761667 on EOCAD (Table S11) was more likely mediated by other risk factors (e.g., hypertension, diabetes, and obesity), rather than dyslipidemia.

The G allele of rs1049673 (Table S9) and rs3211956 (Table S10) significantly decreased LDL-C levels. It indicated that the C allele of rs1049673 and rs3211956 would be associated with higher LDL-C levels. Since LDL-C was considered the major cause of EOCAD and treated as the primary target for therapy [74–76], it indicated that the C allele of rs1049673 and rs3211956 might serve as genetic markers or therapeutic targets for the risk of EOCAD.

In subgroup analyses, the impacts of rs1049673 and rs3211956 on LDL-C levels were significant in Chinese individuals (Table S9 and Table S10), indicating that Chinese individuals with the G allele of rs1049673 and rs3211956 might have a reduced risk of EOCAD. However, whether rs1049673 (Table S12) and rs3211956 (Table S13) affected the risk of EOCAD in other ethnicities, such as individuals of Caucasian and African, was unknown due to the limited number of studies. Therefore, further studies on Caucasian and African individuals are certainly needed. Moreover, the risk of EOCAD increased 3.09-fold in Caucasian individuals with the rs1761667 A allele (Table S11), suggesting that Caucasian individuals with the A allele of rs1761667 were at high risk of EOCAD.

Currently, no genome-wide association study has investigated the impacts of rs1761667, rs1049673, and rs3211956 on lipid profiles or EOCAD risk. However, a genome-wide association study conducted by Elbers et al. [77] indicated that the *CD36* rs3211938 G allele was linked to higher HDL-C levels in African American individuals. Since rs1761667 (Chromosome 7, Position 80082875) [16] and rs3211938 (Chromosome 7, Position 80138385) [77] are located in the same genetic region of the *CD36* gene, it is tempting to speculate that rs1761667 and rs3211938 are very likely with a similar biological function. Indeed, both rs1761667 and rs3211938 reduced CD36 protein expression [16, 78]. Since CD36 protein levels are negatively associated with HDL-C levels [23], it is plausible to observe that the rs1761667 A allele significantly elevated HDL-C levels (Table S8). Moreover, a genome-wide association study by Ellis et al. [79] indicated that the rs3211938 G allele significantly elevated plasma C-reactive protein levels. Since high C-reactive protein levels are closely related to the risk of EOCAD [80–82], it indicates that the impact of rs1761667 on EOCAD risk (Table S11) is at least partly mediated by the impact of rs1761667 on CRP levels. Further studies are needed to confirm this hypothesis.

A previous meta-analysis conducted by Yazdanpanah et al. [83] indicated that the rs1761667 A allele significantly increased HDL-C levels and decreased TG levels. However, moderate to high levels of heterogeneity were observed in TG (*I*^2^ = 47.3%–69.1%), TC (*I*^2^ = 51.4%–89.1%), LDL-C (*I*^2^ = 42.1%–93.5%), and HDL-C (*I*^2^ = 55.0%–72.2%) [83], which largely reduced the preciseness of their conclusions [83]. In contrast, all results were recalculated after excluding studies with heterogeneity (Tables S8–S13), which would no doubt increase the preciseness of conclusions. The present systematic review and meta-analysis had some added value on the same topic. For instance, except rs1761667, this meta-analysis also investigated the impacts of rs1049673 and rs3211956 on lipid profiles and found that the G allele of rs1049673 and rs3211956 significantly reduced LDL-C levels (Table S9 and Table S10). Moreover, this study also investigated the impacts of rs1761667, rs1049673, and rs3211956 on the risk of EOCAD (Tables S11–S13) and found that variants of rs1761667, rs1049673, and rs3211956 might serve as genetic markers for the risk of EOCAD. Importantly, this study demonstrated that *CD36* variants (i.e., rs1761667, rs1049673, and rs3211956) had a significant impact on blood lipid levels, which might increase our understanding of the underlying mechanisms between the *CD36* variants and the risk of EOCAD. At last, this study also indicated that genetic screening of *CD36* variants could be helpful for early intervention or prevention of EOCAD in individuals with high risk factors (e.g., smoking, hypertension, diabetes, and family history of atherosclerotic cardiovascular disease).

One major limitation should be noted when interpreting the results. Other risk factors, such as smoking, hypertension, diabetes, and a family history of atherosclerotic cardiovascular disease, are closely linked to EOCAD onset. However, the present study did not detect the association between *CD36* variants and other risk factors due to the lack of original data from the included studies.

## 5. Conclusions

The rs1761667, rs1049673, and rs3211956 variants of the *CD36* gene have significant impacts on lipid levels and may serve as genetic markers for the risk of EOCAD primarily in Chinese. The impacts of *CD36* variants on EOCAD risk are mediated, at least partly, by dyslipidemia. Genetic screening of *CD36* gene variants may be helpful for early intervention or prevention of EOCAD in individuals with high risk factors.

## Data Availability

The data that support the findings of this study are available from the corresponding author upon reasonable request.

## Supporting Information

Supporting Information Additional supporting information can be found online in the Supporting Information section. Table S1. Characteristics of studies for *CD36* variants. Table S2. Characteristics of studies for rs1761667. Table S3. Characteristics of studies for rs1049673. Table S4. Characteristics of studies for rs3211956. Table S5. Plasma lipid levels by rs1761667. Table S6. Plasma lipid levels by rs1049673. Table S7. Plasma lipid levels by rs3211956. Table S8. Meta-analysis of rs1761667 with lipid levels. Table S9. Meta-analysis of rs1049673 with lipid levels. Table S10. Meta-analysis of rs3211956 with lipid levels. Table S11. Meta-analysis of rs1761667 with EOCAD. Table S12. Meta-analysis of rs1049673 with EOCAD. Table S13. Meta-analysis of rs3211956 with EOCAD. Figure S1. Forest plot for rs1761667 with EOCAD. Figure S2. Forest plot for rs3211956 with EOCAD. Figure S3. Sensitivity analysis for rs1049673 with lipid levels. Figure S4. Sensitivity analysis for rs3211956 with lipid levels. Figure S5. Sensitivity analysis for rs3211956 with EOCAD. Figure S6. Sensitivity analysis for rs1761667 with EOCAD. Figure S7. Sensitivity analysis for rs1761667 with lipid levels. Figure S8. Begg's funnel plot for rs1761667 with lipid levels. Figure S9. Begg's funnel plot for rs1049673 with lipid levels. Figure S10. Begg's funnel plot for rs3211956 with lipid levels. Figure S11. Begg's funnel plot for rs1761667 with EOCAD. Figure S12. Begg's funnel plot for rs1049673 with EOCAD. Figure S13. Begg's funnel plot for rs3211956 with EOCAD.
