# Supplementary material for: Impacts of CD36 Variants on Plasma Lipid Levels and the Risk of Early-Onset Coronary Artery Disease: A Systematic Review and Meta-Analysis
Source: Cardiovasc Ther. 2025 Feb 25;2025:8098173. doi: 10.1155/cdr/8098173 (PMC11879577; doi:10.1155/cdr/8098173)
Supplement: Supporting Information — Additional supporting information can be found online in the Supporting Information section. Table S1. Characteristics of studies for CD36 variants. Table S2. Characteristics of studies for rs1761667. Table S3. Characteristics of studies for rs1049673. Table S4. Characteristics of studies for rs3211956. Table S5. Plasma lipid levels by rs1761667. Table S6. Plasma lipid levels by rs1049673. Table S7. Plasma lipid levels by rs3211956. Table S8. Meta-analysis of rs1761667 with lipid levels. Table S9. Meta-analysis of rs1049673 with lipid levels. Table S10. Meta-analysis of rs3211956 with lipid levels. Table S11. Meta-analysis of rs1761667 with EOCAD. Table S12. Meta-analysis of rs1049673 with EOCAD. Table S13. Meta-analysis of rs3211956 with EOCAD. Figure S1. Forest plot for rs1761667 with EOCAD. Figure S2. Forest plot for rs3211956 with EOCAD. Figure S3. Sensitivity analysis for rs1049673 with lipid levels. Figure S4. Sensitivity analysis for rs3211956 with lipid levels. Figure S5. Sensitivity analysis for rs3211956 with EOCAD. Figure S6. Sensitivity analysis for rs1761667 with EOCAD. Figure S7. Sensitivity analysis for rs1761667 with lipid levels. Figure S8. Begg's funnel plot for rs1761667 with lipid levels. Figure S9. Begg's funnel plot for rs1049673 with lipid levels. Figure S10. Begg's funnel plot for rs3211956 with lipid levels. Figure S11. Begg's funnel plot for rs1761667 with EOCAD. Figure S12. Begg's funnel plot for rs1049673 with EOCAD. Figure S13. Begg's funnel plot for rs3211956 with EOCAD. [file 8098173.f1.doc]

**Table S1.** Characteristics of studies for *CD36* variants.

| **First author, reference** | **Year** | **Country** | **Ethnicity** | **Gender** | **Study population** | **Outcomes** |
| --- | --- | --- | --- | --- | --- | --- |
| Ma et al. 2004 [35] | 2004 | Italy | Caucasian | M | General population | TC/HDL-C |
| Ma et al. 2004 (2) [35] | 2004 | Italy | Caucasian | F | General population | TC/HDL-C |
| Madden et al. 2008 [36] | 2008 | UK | Caucasian | M | General population | TG/LDL-C/HDL-C |
| Lecompte et a. 2011 [37] | 2011 | France | Caucasian | M/F | General population | TC |
| Bayoumy et al. 2012 [38] | 2012 | Egypt | Caucasian | M/F | Patients with metabolic syndrome | TG/TC/LDL-C/HDL-C |
| Zhang et al. 2015 [39] | 2015 | China | Asian | M/F | Patients with ischemic stroke | TG/TC/LDL-C/HDL-C |
| Mrizak et al. 2015 [40] | 2015 | Tunisia | African | F | Patients with obesity | TC/LDL-C |
| Solakivi et al. 2015 [41] | 2015 | Finland | Caucasian | M/F | Patients with hypertension and control subjects | TC |
| Solakivi et al. 2015 (2) [41] | 2015 | Finland | Caucasian | M/F | Patients with hypertension and control subjects | TC |
| Solakivi et al. 2015 (3) [41] | 2015 | Finland | Caucasian | M/F | Patients with hypertension and control subjects | TC |
| Pioltine et al. 2016 [42] | 2016 | Brazil | Brazilian | M/F | Patients with obesity | LDL-C |
| Ramos-Lopez et al. 2016 [43] | 2016 | Mexico | Mexican | M/F | Patients with chronic hepatitis C | TG/TC/LDL-C/HDL-C |
| Momeni-Moghaddam et al. 2019 [32] | 2019 | Iran | Caucasian | M/F | Patients with CAD | TG/TC/LDL-C/HDL-C |
| Momeni-Moghaddam et al. 2019 (2) [32] | 2019 | Iran | Caucasian | M/F | Patients with hypertension | TG/TC/LDL-C/HDL-C |
| Momeni-Moghaddam et al. 2019 (3) [32] | 2019 | Iran | Caucasian | M/F | Patients with CAD and hypertension | TG/TC/LDL-C/HDL-C |
| Momeni-Moghaddam et al. 2019 (4) [32] | 2019 | Iran | Caucasian | M/F | General population | TG/TC/LDL-C/HDL-C |
| Fakhry et al. 2020 [44] | 2020 | Egypt | Caucasian | F | Patients with preeclampsia | TG/TC |
| Du et al. 2020 [45] | 2020 | China | Asian | M/F | Patients with EOCAD and control subjects | TG/TC/LDL-C/HDL-C |
| Che et al. 2014 [33] | 2014 | China | Asian | M/F | Patients with EOCAD and control subjects | LDL-C |
| Wang et al. 2012 [46] | 2012 | China | Asian | M/F | Patients with hypertension | TG/TC/LDL-C/HDL-C |
| Wang et al. 2015 [47] | 2015 | China | Asian | M/F | Patients with EOCAD and control subjects | TG/TC/LDL-C/HDL-C |
| Shao et al. 2012 [48] | 2012 | China | Asian | M/F | Patients with EOCAD and control subjects | TG/TC/LDL-C/HDL-C |
| Heni et al. 2011 [49] | 2011 | Germany | Caucasian | M/F | Patients with T2DM and control subjects | TG/TC/LDL-C/HDL-C |
| Zhao et al. 2014 [50] | 2014 | China | Asian | M/F | Patients with EOCAD and control subjects | TG/TC/LDL-C/HDL-C |
| Yang QQ [54] | 2020 | China | Asian | M/F | Patients with T2DM and control subjects | TG/TC/LDL-C/HDL-C |
| Touré et al. 2022 [23] | 2022 | Dakar | African | F | Patients with T2DM | TG/TC/LDL-C/HDL-C |
| Touré et al. 2022 [23] | 2022 | Dakar | African | F | General population | TG/TC/LDL-C/HDL-C |
| Touré et al. 2022 [24] | 2022 | Dakar | African | F | Patients with obesity | TG/TC/LDL-C/HDL-C |
| Touré et al. 2022 [24] | 2022 | Dakar | African | F | Patients with Obese diabetic | TG/TC/LDL-C/HDL-C |
| Takahashi M [55] | 2022 | Japan | Asian | F | General population | TC |

M: male; F: female; CAD: coronary artery disease; EOCAD: early-onset coronary artery disease; T2DM: type 2 diabetes mellitus; TG: triglycerides; TC: total cholesterol; LDL-C: low-density lipoprotein cholesterol; HDL-C: high-density lipoprotein cholesterol.

**Table S2.** Characteristics of studies for rs1761667.

| **First author, reference** | **Year** | **Country** | **Ethnicity** | **Gender** | **Sample size**  **(case/control)** | **Case (N)** | | | |  | **Control (N)** | | | | ***P*_HWE_** |
| --- | --- | --- | --- | --- | --- | --- | --- | --- | --- | --- | --- | --- | --- | --- | --- |
|  |  |  |  |  |  | **GG** | **GA** | **AA** | **A** |  | **GG** | **GA** | **AA** | **A** |  |
| Momeni-Moghaddam et al. 2019 [39] | 2019 | Iran | Caucasian | M/F | 65/65 | 12 | 45 | 8 | 0.469 |  | 26 | 30 | 9 | 0.369 | 0.94 |
| Boghdady et al. 2016 [51] | 2016 | Egypt | Caucasian | M/F | 47/27 | 9 | 30 | 8 | 0.489 |  | 12 | 5 | 10 | 0.463 | 0.00 |
| Ding et al. 2017 [52] | 2017 | China | Asian | M/F | 119/216 | 39 | 67 | 13 | 0.390 |  | 92 | 89 | 35 | 0.368 | 0.09 |

EOCAD: early-onset coronary artery disease.

**Table S3.** Characteristics of studies for rs1049673.

| **First author, reference** | **Year** | **Country** | **Ethnicity** | **Gender** | **Sample size**  **(case/control)** | **Case (N)** | | | |  | **Control (N)** | | | | ***P*_HWE_** |
| --- | --- | --- | --- | --- | --- | --- | --- | --- | --- | --- | --- | --- | --- | --- | --- |
|  |  |  |  |  |  | **CC** | **CG** | **GG** | **G** |  | **CC** | **CG** | **GG** | **G** |  |
| Che et al. 2014 [33] | 2014 | China | Asian | M/F | 79/56 | 18 | 41 | 20 | 0.512 |  | 9 | 26 | 21 | 0.607 | 0.84 |
| Wang et al. 2015 [47] | 2015 | China | Asian | M/F | 186/151 | 62 | 93 | 31 | 0.416 |  | 35 | 70 | 46 | 0.536 | 0.40 |
| Shao et al. 2012 [48] | 2012 | China | Asian | M/F | 79/56 | 18 | 41 | 20 | 0.512 |  | 9 | 26 | 21 | 0.607 | 0.84 |
| Rać et al. 2013 [53] | 2013 | Poland | Caucasian | M/F | 206/123 | 48 | 113 | 45 | 0.493 |  | 40 | 57 | 26 | 0.443 | 0.50 |

EOCAD: early-onset coronary artery disease.

**Table S4.** Characteristics of studies for rs3211956.

| **First author, reference** | **Year** | **Country** | **Ethnicity** | **Gender** | **Sample size**  **(case/control)** | **Case (N)** | | | |  | **Control (N)** | | | | ***P*_HWE_** |
| --- | --- | --- | --- | --- | --- | --- | --- | --- | --- | --- | --- | --- | --- | --- | --- |
|  |  |  |  |  |  | TT | TG | GG | G |  | TT | TG | GG | G |  |
| Che et al. 2014 [33] | 2014 | China | Asian | M/F | 79/54 | 44 | 27 | 8 | 0.272 |  | 22 | 28 | 4 | 0.333 | 0.22 |
| Wang et al. 2015 [47] | 2015 | China | Asian | M/F | 186/151 | 125 | 55 | 6 | 0.180 |  | 89 | 49 | 13 | 0.248 | 0.11 |
| Zhao et al. 2014 [50] | 2014 | China | Asian | M/F | 102/72 | 62 | 32 | 8 | 0.235 |  | 30 | 38 | 4 | 0.319 | 0.07 |
| Rać et al. 2013 [53] | 2013 | Poland | Caucasian | M/F | 206/123 | 124 | 74 | 8 | 0.218 |  | 84 | 33 | 6 | 0.183 | 0.26 |

EOCAD: early-onset coronary artery disease.

**Table S5.** Plasma lipid levels by rs1761667.

| **First author, reference** | **Number** | | |  | | **TG, mmol/L** | | |  | | **TC, mmol/L** | | | |  | | **LDL-C, mmol/L** | | |  | **HDL-C, mmol/L** | | | |  |
| --- | --- | --- | --- | --- | --- | --- | --- | --- | --- | --- | --- | --- | --- | --- | --- | --- | --- | --- | --- | --- | --- | --- | --- | --- | --- |
|  | **GG** | **GA+AA** | **A** | |  | | **GG** | **GA+AA** | |  | | **GG** | **GA+AA** | | |  | | **GG** | **GA+AA** | |  | **GG** | **GA+AA** | | |
| Ma et al. 2004 [35] | 45 | 169 | 0.544392523 | |  | | - | - | |  | | 5.07±1.06 | 5.07±1.11 | | |  | | - | - | |  | 1.11±0.23 | 1.21±0.31 | | |
| Ma et al. 2004 (2) [35] | 70 | 258 | 0.530487805 | |  | | - | - | |  | | 4.78±1.06 | 4.99±0.96 | | |  | | - | - | |  | 1.45±0.34 | 1.50±0.33 | | |
| Madden et al. 2008 [36] | 27 | 81 | 0.513888889 | |  | | 1.54±0.85 | 1.46±0.78 | |  | | - |  | - | |  | | 4.57±1.05 | 4.36±0.87 | |  | 1.29±0.30 | 1.27±0.25 | | |
| Lecompte et a. 2011 [37] | 218 | 745 | 0.523883697 | |  | |  |  | |  | | 5.41±0.85 | 5.57±0.91 | | |  | |  |  | |  | - |  | - | |
| Bayoumy et al. 2012 [38] | 5 | 95 | 0.6 | |  | | 3.16±0.56 | 2.48±0.44 | |  | | 6.08±1.03 | 5.58±0.85 | | |  | | 4.4±0.78 | 3.44±1.04 | |  | 0.96±0.13 | 1.07±0.14 | | |
| Zhang et al. 2015[39] | 543 | 816 | 0.368285504 | |  | | 1.11±0.71 | 1.13±0.71 | |  | | 5.02±0.04 | 5.07±0.05 | | |  | | 3.03±0.04 | 3.09±0.04 | |  | 1.38±0.04 | 1.37±0.02 | | |
| Mrizak et al. 2015 [40] | 42 | 161 | 0.541871921 | |  | | - | - | |  | | 5.2±0 | 5.06±0.34 | | |  | | 3.18±0 | 3.02±0.29 | |  | - |  | - | |
| Solakivi et al. 2015 [41] | 158 | 578 | 0.529891304 | |  | | - | - | |  | | 5.42±1.08 | 5.37±0.96 | | |  | | - | - | |  | - |  | - | |
| Solakivi et al. 2015 (2) [41] | 145 | 530 | 0.528148148 | |  | | - | - | |  | | 5.59±1.22 | 5.48±1.05 | | |  | | - | - | |  | - |  | - | |
| Solakivi et al. 2015 (3) [41] | 129 | 463 | 0.521114865 | |  | | - | - | |  | | 5.72±1.04 | 5.59±1.13 | | |  | | - | - | |  | - |  | - | |
| Pioltine et al. 2016 [42] | 121 | 345 | - | |  | | - | - | |  | | - |  | - | |  | | 2.66±0.73 | 2.65±0.80 | |  | - |  | - | |
| Ramos-Lopez et al. 2016 [43] | 11 | 62 | 0.575342466 | |  | | 1.61±0.58 | 1.48±0.64 | |  | | 4.08±1.32 | 4.05±1.07 | | |  | | 2.61±1.1 | 2.36±0.99 | |  | 0.87±0.25 | 1.06±0.36 | | |
| Momeni-Moghaddam et al. 2019 [32] | 12 | 53 | 0.469230769 | |  | | 1.21±0.49 | 1.25±0.69 | |  | | 3.61±1 | 3.76±1.04 | | |  | | 2.07±0.91 | 2.19±0.88 | |  | 0.99±0.16 | 1.01±0.29 | | |
| Momeni-Moghaddam et al. 2019 (2) [32] | 6 | 46 | 0.5 | |  | | 1.02±0.26 | 1.35±0.69 | |  | | 3.73±0.63 | 3.69±0.88 | | |  | | 2.13±0.69 | 2.05±0.82 | |  | 1.13±0.14 | 0.97±0.26 | | |
| Momeni-Moghaddam et al. 2019 (3) [32] | 17 | 40 | 0.368421053 | |  | | 1.64±0.91 | 1.06±0.36 | |  | | 3.75±0.93 | 3.43±0.85 | | |  | | 2.12±0.72 | 1.95±0.72 | |  | 0.88±0.17 | 1±0.27 | | |
| Momeni-Moghaddam et al. 2019 (4) [32] | 26 | 39 | 0.369230769 | |  | | 1.27±0.45 | 1.19±0.42 | |  | | 3.92±1.26 | 3.84±1.17 | | |  | | 2.28±1.14 | 2.10±1.04 | |  | 1.06±0.32 | 1.1±0.23 | | |
| Fakhry et al. 2020 [44] | 22 | 78 | 0.51 | |  | | 2.37±0.23 | 2.50±0.32 | |  | | 5.24±1.41 | 5.46±1.28 | | |  | | - | - | |  | - |  | - | |
| Yang et al. 2020 [54] | 71 | 110 | - | |  | | 2.11±1.93 | 2.01±1.43 | |  | | 4.41±1.16 | 4.63±1.05 | | |  | | 2.28±0.72 | 2.55±0.91 | |  | 1.05±0.30 |  | 1.09±0.34 | |
| Touré et al. 2022 [23] | 25 | 25 | - | |  | | 0.95±0.63 | 0.92±0.47 | |  | | 2.28±0.42 | 2.29±0.58 | | |  | | 1.62±0.47 | 1.69±0.59 | |  | 0.59±0.17 | 0.54±0.18 | 0.54±0.18 | |
| Touré et al. 2022 [23] | 24 | 26 | - | |  | | 0.84±0.35 | 0.76±0.28 | |  | | 2.32±0.42 | 2.04±0.45 | | |  | | 1.46±0.30 | 1.34±0.41 | |  | 0.63±0.13 | 0.61±0.15 | 0.61±0.15 | |
| Touré et al. 2022 [24] | 21 | 29 | - | |  | | 0.86±0.43 | 0.73±0.24 | |  | | 2.12±0.43 | 2.07±0.40 | | |  | | 1.38±0.40 | 1.37±0.41 | |  | 0.58±0.16 |  | 0.58±0.11 | |
| Touré et al. 2022 [24] | 18 | 32 | - | |  | | 0.84±0.36 | 0.89±0.39 | |  | | 2.37±0.65 | 2.19±0.49 | | |  | | 1.68±0.60 | 1.62±0.49 | |  | 0.64±0.19 |  | 0.54±0.12 | |
| Takahashi et al. 2022 [55] | 32 | 29 | - | |  | | - | - | |  | | 5.08±0.70 | 5.23±0.76 | | |  | | - | - | |  | - |  | - | |

TG: triglycerides; TC: total cholesterol; LDL-C: low-density lipoprotein cholesterol; HDL-C: high-density lipoprotein cholesterol.

**Table S6.** Plasma lipid levels by rs1049673.

| **First author, reference** | **Number** | | |  | **TG, mmol/L** | | |  | **TC, mmol/L** | |  | **LDL-C, mmol/L** | | |  | **HDL-C, mmol/L** | | |
| --- | --- | --- | --- | --- | --- | --- | --- | --- | --- | --- | --- | --- | --- | --- | --- | --- | --- | --- |
|  | **CC** | **CG+GG** | **G** |  | **CC** | **CG+GG** | |  | **CC** | **CG+GG** |  | **CC** | **CG+GG** | |  | **CC** | **CG+GG** | |
| Du et al. 2020 [45] | 45 | 133 | - |  | 2.207±0.34  (0.34) | 1.91±1.27 | |  | 4.544±0.08  (0.08) | 5.01±0.92 |  | 2.796±0.10  (0.10) | 3.28±0.81 | |  | 0.911±0.090  (0.09) | 0.93±0.35 | |
| Madden et al. 2008 [36] | 26 | 82 | 0.49 |  | 1.50±0.74 | 1.47±0.81 | |  | - | - |  | 4.41±0.94 | 4.4±0.91 | |  | 1.25±0.24 | 1.27±0.27 | |
| Che et al. 2014 [33] | 27 | 108 | 0.551851852 |  | - |  | - |  | - | - |  | 3.3±0.7 | 2.96±0.76 | |  | - |  | - |
| Wang et al. 2012 [46] | 284 | 841 | 0.494222222 |  | 2.1±1.3 | 2.1±1.33 | |  | 4.8±0.9 | 4.83±1.0 |  | 2.8±0.7 | 2.83±0.8 | |  | 1.21±0.28 | 1.17±0.31 | |
| Lecompte et al. 2011 [37] | 203 | 789 | 0.544310171 |  | - |  | - |  | 5.45±0.88 | 5.56±0.88 |  | - |  | - |  | - |  | - |
| Ma et al. 2004 [35] | 37 | 168 | 0.548780488 |  | - |  | - |  | 5.2±1.01 | 5.04±1.13 |  | - |  | - |  | 1.19±0.28 | 1.19±0.32 | |
| Ma et al. 2004 (2) [35] | 67 | 246 | 0.547923323 |  | - |  | - |  | 5.07±0.98 | 4.89±0.95 |  | - |  | - |  | 1.47±0.31 | 1.49±0.32 | |
| Wang et al. 2015 [47] | 97 | 240 | 0.470326409 |  | 2.49±2.43 | 2.30±1.61 | |  | 4.91±1.10 | 4.91±1.31 |  | 3.49±1.14 | 3.09±1.04 | |  | 1.06±0.29 | 1.14±0.37 | |
| Shao et al. 2012 [48] | 162 | 378 | - |  | 1.85±1.25 | 2.11±1.7 | |  | 5.3±0.95 | 4.94±0.96 |  | 3.33±0.73 | 2.96±0.72 | |  | 1.17±0.27 | 1.15±0.29 | |

TG: triglycerides; TC: total cholesterol; LDL-C: low-density lipoprotein cholesterol; HDL-C: high-density lipoprotein cholesterol.

**Table S7.** Plasma lipid levels by rs3211956.

| **First author, reference** | **Number** | | |  | **TG, mmol/L** | |  | **TC, mmol/L** | | |  | **LDL-C, mmol/L** | |  | **HDL-C, mmol/L** | | |
| --- | --- | --- | --- | --- | --- | --- | --- | --- | --- | --- | --- | --- | --- | --- | --- | --- | --- |
|  | **TT** | **TG+GG** | **G** |  | **TT** | **TG+GG** |  | **TT** | **TG+GG** | |  | **TT** | **TG+GG** |  | **TT** | **TG+GG** | |
| Du et al. 2020 [45] | 109 | 69 | - |  | 1.978±0.30  (0.30) | 1.98±0.30  (0.30) |  | 4.903±0.09  (0.09) | 4.87±0.12 | |  | 3.121±0.13  (0.13) | 3.2±0.12  (0.12) |  | 0.958±0.1  (0.1) | 0.88±0.1  (0.1) | |
| Che et al. 2014 [33] | 66 | 67 | 0.296992481 |  | - | - |  | - |  | - |  | 3.23 ± 0.78 | 2.87±0.64 |  | - |  | - |
| Heni et al. 2011 [49] | 1406 | 263 | 0.07915493 |  | 1.46±1.73 | 1.25±0.87 |  | 4.97±1.01 | 4.91±0.92 | |  | 3.08±0.85 | 3.06±0.6 |  | 1.4±0.36 | 1.45±0.39 | |
| Zhao et al. 2014 [50] | 92 | 82 | 0.270114943 |  | 2.05±1.40 | 2.05±1.87 |  | 5.15±1.05 | 4.93±0.88 | |  | 2.72±1.14 | 2.67±0.87 |  | 1.36±0.62 | 1.54±0.73 | |
| Wang et al. 2015 [47] | 214 | 123 | 0.210682493 |  | 2.36±2.07 | 1.67±1.50 |  | 4.97±1.15 | 4.8±1.41 | |  | 3.29±1.06 | 3.06±1.11 |  | 1.11±0.31 | 1.13±0.42 | |

TG: triglycerides; TC: total cholesterol; LDL-C: low-density lipoprotein cholesterol; HDL-C: high-density lipoprotein cholesterol.

**Table S8. Meta-analysis of rs1761667 with lipid levels.**

| **Groups or subgroups** | ***P*_H_** | **SMD (95% CI)** | ***P*_SMD_** |  | **Groups or subgroups** | ***P*_H_** | **SMD (95% CI)** | ***P*_SMD_** |
| --- | --- | --- | --- | --- | --- | --- | --- | --- |
| ***Overall results*** | | | |  | ***Recalculated results that eliminated heterogeneity*** | | | |
| **TG** |  |  |  |  | **TG** |  |  |  |
| All | 0.01 | -0.13 (-0.32-0.06)  04 | 0.18 |  | All | 0.66 | 0.01 (-0.08-0.09)  04 | 0.88 |
| Studies in HWE | 0.37 | 0.02 (-0.10-0.15) | 0.72 |  | Studies in HWE | 0.37 | 0.02 (-0.10-0.15) | 0.72 |
| Caucasian | <0.001 | -0.22 (-0.68-0.23) | 0.34 |  | Caucasian | 0.32 | 0.09 (-0.18-0.35) | 0.51 |
| CAD | 0.02 | -0.48 (-1.52-0.57) | 0.37 |  | CAD | 0.48 | 0.00 (-0.13-0.13) | 0.94 |
| General population | 0.80 | -0.14 (-0.47-0.19) | 0.41 |  | General population | 0.80 | -0.14 (-0.47-0.19) | 0.41 |
| **TC** |  |  |  |  | **TC** |  |  |  |
| All | <0.001 | 0.02 (-0.23-0.27)  04 | 0.90 |  | All | 0.21 | 0.05 (-0.04-0.13)  04 | 0.28 |
| Studies in HWE | <0.001 | 0.14 (-0.22-0.49) | 0.45 |  | Studies in HWE | 0.20 | 0.02 (-0.06-0.10) | 0.59 |
| Caucasian | 0.20 | 0.01 (-0.09-0.10)  04 | 0.93 |  | Caucasian | 0.20 | 0.01 (-0.06-0.09) | 0.75 |
| Female | 0.87 | 0.20 (-0.03-0.43) | 0.09 |  | Female | 0.87 | 0.20 (-0.03-0.43) | 0.09 |
| CAD | 0.24 | -0.13 (-0.63-0.37) | 0.62 |  | CAD | 0.24 | -0.13 (-0.56-0.29) | 0.53 |
| General population | 0.60 | 0.15 (0.03-0.27) | 0.01 |  | General population | 0.60 | 0.15 (0.03-0.27) | 0.01 |
| **LDL-C** |  |  |  |  | **LDL-C** |  |  |  |
| All | <0.001 | -0.00 (-0.50-0.50)  04 | 0.99 |  | All | 0.39 | -0.03 (-0.15-0.09)  04 | 0.65 |
| Studies in HWE | <0.001 | 0.23 (-0.93-1.39) | 0.70 |  | Studies in HWE | 0.98 | -0.21 (-0.50-0.08) | 0.16 |
| Caucasian | 0.59 | -0.20 (-0.44-0.04) | 0.10 |  | Caucasian | 0.59 | -0.20 (-0.44-0.04) | 0.10 |
| CAD | 0.39 | -0.07 (-0.49-0.35) | 0.75 |  | CAD | 0.39 | -0.07 (-0.49-0.35) | 0.75 |
| General population | 0.85 | -0.20 (-0.53-0.13) | 0.23 |  | General population | 0.85 | -0.20 (-0.53-0.13) | 0.23 |
| **HDL-C** |  |  |  |  | **HDL-C** |  |  |  |
| All | <0.001 | 0.02 (-0.18-0.22)  04 | 0.83 |  | All | 0.33 | 0.13 (0.01-0.26)  04 | 0.04 |
| Studies in HWE | <0.001 | 0.09 (-0.23-0.40) | 0.58  18 |  | Studies in HWE | 0.33 | 0.13 (0.01-0.26)  04 | 0.04 |
| Caucasian | 0.27 | 0.17 (-0.01-0.36) | 0.07 |  | Caucasian | 0.46 | 0.24 (0.17-0.31) | 0.03 |
| Male | 0.14 | 0.16 (-0.25-0.56) | 0.45 |  | Male | - | - | - |
| CAD | 0.59 | 0.07 (-0.06-0.21) | 0.29 |  | CAD | - | - | - |
| General population | 0.52 | 0.17 (-0.01-0.34) | 0.06 |  | General population | 0.51 | 0.02 (-0.31-0.35) | 0.90 |

SMD: standardized mean difference; 95% CI: 95% confidence interval; *P*_H_: *P*_Heterogeneity_; HWE: Hardy-Weinberg equilibrium; TG:

triglycerides; TC: total cholesterol; LDL-C: low-density lipoprotein cholesterol; HDL-C: high-density lipoprotein cholesterol.

**Table S9. Meta-analysis of rs1049673 with lipid levels.**

| **Groups or subgroups** | ***P*_H_** | **SMD (95% CI)** | ***P*_SMD_** |  | **Groups or subgroups** | ***P*_H_** | **SMD (95% CI)** | ***P*_SMD_** |
| --- | --- | --- | --- | --- | --- | --- | --- | --- |
| ***Overall results*** | | | |  | ***Recalculated results that eliminated heterogeneity*** | | | |
| **TG** |  |  |  |  | **TG** |  |  |  |
| All | 0.19 | 0.00 (-0.09-0.09)  04 | 0.93 |  | All | 0.19 | 0.00 (-0.09-0.10)  04 | 0.92 |
| Studies in HWE | 0.52 | -0.05 (-0.16-0.06)  04 | 0.36 |  | Studies in HWE | 0.52 | -0.05 (-0.16-0.06)  04 | 0.36 |
| Asian | 0.11 | 0.01 (-0.09-0.10)  04 | 0.90 |  | Asian | 0.11 | 0.01 (-0.09-0.10)  04 | 0.90 |
| **TC** |  |  |  |  | **TC** |  |  |  |
| All | <0.001 | -0.01 (-0.20-0.18)  04 | 0.94 |  | All | 0.30 | 0.02 (-0.06-0.11)  04 | 0.59 |
| Studies in HWE | 0.01 | 0.06 (-0.03-0.14)  04 | 0.18 |  | Studies in HWE | 0.30 | 0.02 (-0.06-0.11)  04 | 0.59 |
| Caucasian | 0.09 | -0.04 (-0.26-0.19) | 0.75 |  | Caucasian | 0.09 | 0.02 (-0.10-0.15) | 0.71 |
| Asian | <0.001 | 0.03 (-0.27-0.34) | 0.83 |  | Asian | 0.82 | 0.02 (-0.09-0.14)  04 | 0.70 |
| General population | 0.09 | -0.04 (-0.26-0.19) | 0.75 |  | General population | 0.09 | 0.02 (-0.10-0.15) | 0.71 |
| **LDL-C** |  |  |  |  | **LDL-C** |  |  |  |
| All | <0.001 | -0.11 (-0.43-0.21)  04 | 0.50 |  | All | 0.22 | -0.42 (-0.55--0.29)  04 | <0.001 |
| Studies in HWE | <0.001 | -0.02 (-0.36-0.32)  04 | 0.91 |  | Studies in HWE | 0.29 | -0.32 (-0.51--0.14)  04 | <0.01 |
| Asian | <0.001 | -0.13 (-0.49-0.24)  04 | 0.50 |  | Asian | 0.67 | -0.46 (-0.60--0.32)  04 | <0.001 |
| **HDL-C** |  |  |  |  | **HDL-C** |  |  |  |
| All | 0.24 | -0.03 (-0.11-0.06)  04 | 0.56 |  | All | 0.24 | -0.03 (-0.11-0.06)  04 | 0.56 |
| Studies in HWE | 0.17 | -0.01 (-0.11-0.08)  04 | 0.79 |  | Studies in HWE | 0.17 | -0.01 (-0.11-0.08)  04 | 0.79 |
| Caucasian | 0.95 | 0.05 (-0.15-0.24) | 0.64 |  | Caucasian | 0.95 | 0.05 (-0.15-0.24) | 0.64 |
| Asian | 0.07 | -0.04 (-0.13-0.05)  04 | 0.38 |  | Asian | 0.07 | -0.04 (-0.13-0.05)  04 | 0.38 |
| General population | 0.95 | 0.05 (-0.15-0.24) | 0.64 |  | General population | 0.95 | 0.05 (-0.15-0.24) | 0.64 |

SMD: standardized mean difference; 95% CI: 95% confidence interval; *P*_H_: *P*_Heterogeneity_; HWE: Hardy-Weinberg equilibrium;

TG: triglycerides; TC: total cholesterol; LDL-C: low-density lipoprotein cholesterol; HDL-C: high-density lipoprotein cholesterol.

**Table S10. Meta-analysis of rs3211956 with lipid levels.**

| **Groups or subgroups** | ***P*_H_** | **SMD (95% CI)** | ***P*_SMD_** |  | **Groups or subgroups** | ***P*_H_** | **SMD (95% CI)** | ***P*_SMD_** |
| --- | --- | --- | --- | --- | --- | --- | --- | --- |
| ***Overall results*** | | | |  | ***Recalculated results that eliminated heterogeneity*** | | | |
| **TG** |  |  |  |  | **TG** |  |  |  |
| All | 0.13 | -0.15 (-0.25--0.05)  04 | <0.01 |  | All | 0.13 | -0.15 (-0.25--0.05)  04 | <0.01 |
| Studies in HWE | 0.13 | -0.15 (-0.25--0.05)  04 | <0.01 |  | Studies in HWE | 0.13 | -0.15 (-0.25--0.05)  04 | <0.01 |
| Asian | 0.06 | -0.17 (-0.33--0.02)  04 | 0.03 |  | Asian | 0.06 | -0.17 (-0.33--0.02)  04 | 0.03 |
| **TC** |  |  |  |  | **TC** |  |  |  |
| All | 0.40 | -0.12 (-0.22--0.02)  04 | 0.02 |  | All | 0.40 | -0.12 (-0.22--0.02)  04 | 0.02 |
| Studies in HWE | 0.40 | -0.12 (-0.22--0.02)  04 | 0.02 |  | Studies in HWE | 0.40 | -0.12 (-0.22--0.02)  04 | 0.02 |
| Asian | 0.62 | -0.21 (-0.36--0.05) | 0.01 |  | Asian | 0.62 | -0.21 (-0.36--0.05) | 0.01 |
| **LDL-C** |  |  |  |  | **LDL-C** |  |  |  |
| All | <0.001 | -0.03 (-0.32-0.25)  04 | 0.83 |  | All | 0.06 | -0.11 (-0.21--0.01)  04 | 0.04 |
| Studies in HWE | <0.001 | -0.03 (-0.32-0.25)  04 | 0.83 |  | Studies in HWE | 0.06 | -0.11 (-0.21--0.01)  04 | 0.04 |
| Asian | <0.001 | -0.03 (-0.47-0.41)  04 | 0.88 |  | Asian | 0.14 | -0.23 (-0.39--0.07)  04 | 0.01 |
| **HDL-C** |  |  |  |  | **HDL-C** |  |  |  |
| All | <0.001 | -0.07 (-0.43-0.30)  04 | 0.72 |  | All | 0.54 | 0.13 (0.03-0.24)  04 | 0.01 |
| Studies in HWE | 0.17 | -0.01 (-0.11-0.08)  04 | 0.79 |  | Studies in HWE | 0.54 | 0.13 (0.03-0.24)  04 | 0.01 |
| Asian | <0.001 | -0.15 (-0.72-0.43)  04 | 0.61 |  | Asian | 0.27 | 0.13 (-0.05-0.31)  04 | 0.15 |

SMD: standardized mean difference; 95% CI: 95% confidence interval; *P*_H_: *P*_Heterogeneity_; HWE: Hardy-Weinberg equilibrium;

TG: triglycerides; TC: total cholesterol; LDL-C: low-density lipoprotein cholesterol; HDL-C: high-density lipoprotein cholesterol.

**Table S11. Meta-analysis of rs1761667 with EOCAD.**

| **Groups or subgroups** | ***P*_H_** | **OR (95% CI)** | ***P*_OR_** |  | **Groups or subgroups** | ***P*_H_** | **OR (95% CI)** | ***P*_OR_** |
| --- | --- | --- | --- | --- | --- | --- | --- | --- |
| ***Overall results*** | | | |  | ***Recalculated results that eliminated heterogeneity*** | | | |
| Allelic model (A vs. G) |  |  |  |  | Allelic model (A vs. G) |  |  |  |
| All | 0.56 | 1.20 (0.93-1.54)  04 | 0.16 |  | All | 0.56 | 1.20 (0.93-1.54)  04 | 0.16 |
| Studies in HWE | 0.30 | 1.21 (0.92-1.59)  04 | 0.17 |  | Studies in HWE | 0.30 | 1.21 (0.92-1.59)  04 | 0.17 |
| Caucasian | 0.47 | 1.36 (0.91-2.02)  04 | 0.13 |  | Caucasian | 0.47 | 1.36 (0.91-2.02)  04 | 0.13 |
| Additive model (AA vs. GG) |  |  |  |  | Additive model (AA vs. GG) |  |  |  |
| All | 0.54 | 1.09 (0.62-1.89)  04 | 0.77 |  | All | 0.54 | 1.09 (0.62-1.89)  04 | 0.77 |
| Studies in HWE | 0.27 | 1.09 (0.59-2.02)  04 | 0.78 |  | Studies in HWE | 0.27 | 1.09 (0.59-2.02)  04 | 0.78 |
| Caucasian | 0.50 | 1.46 (0.62-3.45)  04 | 0.39 |  | Caucasian | 0.50 | 1.46 (0.62-3.45)  04 | 0.39 |
| Dominant model (GA+AA vs. GG) |  |  |  |  | Dominant model (GA+AA vs. GG) |  |  |  |
| All | 0.21 | 1.95 (1.34-2.84)  04 | <0.001 |  | All | 0.21 | 1.95 (1.34-2.84)  04 | <0.001 |
| Studies in HWE | 0.16 | 1.81 (1.21-2.70)  04 | <0.01 |  | Studies in HWE | 0.16 | 1.81 (1.21-2.70)  04 | <0.01 |
| Caucasian | 0.84 | 3.09 (1.63-5.84)  04 | <0.01 |  | Caucasian | 0.84 | 3.09 (1.63-5.84)  04 | <0.01 |
| Recessive model (GG+GA vs. AA) |  |  |  |  | Recessive model (GG+GA vs. AA) |  |  |  |
| All | 0.47 | 1.65 (1.00-2.72)  04 | 0.05 |  | All | 0.47 | 1.65 (1.00-2.72)  04 | 0.05 |
| Studies in HWE | 0.61 | 1.44 (0.82-2.52  04 | 0.21 |  | Studies in HWE | 0.61 | 1.44 (0.82-2.52  04 | 0.21 |
| Caucasian | 0.23 | 1.74 (0.83-3.66)  04 | 0.14 |  | Caucasian | 0.23 | 1.74 (0.83-3.66)  04 | 0.14 |

EOCAD: early-onset coronary artery disease; SMD: standardized mean difference; 95% CI: 95% confidence interval; *P*_H_: *P*_Heterogeneity_; HWE: Hardy-Weinberg equilibrium; TG: triglycerides; TC: total cholesterol; LDL-C: low-density lipoprotein cholesterol; HDL-C: high-density lipoprotein cholesterol.

**Table S12. Meta-analysis of rs1049673 with EOCAD.**

| **Groups or subgroups** | ***P*_H_** | **OR (95% CI)** | ***P*_OR_** |  | **Groups or subgroups** | ***P*_H_** | **OR (95% CI)** | ***P*_OR_** |
| --- | --- | --- | --- | --- | --- | --- | --- | --- |
| ***Overall results*** | | | |  | ***Recalculated results that eliminated heterogeneity*** | | | |
| Allelic model (G vs. C) |  |  |  |  | Allelic model (G vs. C) |  |  |  |
| All | 0.02 | 0.78 (0.54-1.12)  04 | 0.18 |  | All | 0.92 | 0.64 (0.51-0.81)  04 | <0.001 |
| Studies in HWE | 0.02 | 0.78 (0.54-1.12)  04 | 0.18 |  | Studies in HWE | 0.22 | 0.64 (0.51-0.81)  04 | <0.001 |
| Asian | 0.92 | 0.64 (0.51-0.81) | <0.001 |  | Asian | 0.22 | 0.64 (0.51-0.81)  04 | <0.001 |
| Additive model (GG vs. CC) |  |  |  |  | Additive model (GG vs. CC) |  |  |  |
| All | 0.02 | 0.61 (0.30-1.24)  04 | 0.17 |  | All | 0.90 | 0.42 (0.26-0.67)  04 | <0.001 |
| Studies in HWE | 0.02 | 0.61 (0.30-1.24)  04 | 0.17 |  | Studies in HWE | 0.90 | 0.42 (0.26-0.67)  04 | <0.001 |
| Asian | 0.90 | 0.42 (0.26-0.67)  04 | <0.001 |  | Asian | 0.90 | 0.42 (0.26-0.67)  04 | <0.001 |
| Dominant model (CG+GG vs. CC) |  |  |  |  | Dominant model (CG+GG vs. CC ) |  |  |  |
| All | 0.04 | 0.83 (0.48-1.44)  04 | 0.51 |  | All | 0.89 | 0.61 (0.40-0.94)  04 | 0.02 |
| Studies in HWE | 0.04 | 0.83 (0.48-1.44)  04 | 0.51 |  | Studies in HWE | 0.89 | 0.61 (0.40-0.94)  04 | 0.02 |
| Asian | 0.89 | 0.61 (0.40-0.94)  04 | 0.02 |  | Asian | 0.89 | 0.61 (0.40-0.94)  04 | 0.02 |
| Recessive model (CG+CC vs. GG) |  |  |  |  | Recessive model (CG+CC vs. GG) |  |  |  |
| All | 0.85 | 0.51 (0.35-0.73)  04 | <0.001 |  | All | 0.85 | 0.51 (0.35-0.73)  04 | <0.001 |
| Studies in HWE | 0.85 | 0.51 (0.35-0.73)  04 | <0.001 |  | Studies in HWE | 0.85 | 0.51 (0.35-0.73)  04 | <0.001 |
| Asian | 0.85 | 0.51 (0.35-0.73)  04 | <0.001 |  | Asian | 0.85 | 0.51 (0.35-0.73)  04 | <0.001 |

EOCAD: early-onset coronary artery disease; SMD: standardized mean difference; 95% CI: 95% confidence interval; *P*_H_: *P*_Heterogeneity_; HWE: Hardy-Weinberg equilibrium; TG: triglycerides; TC: total cholesterol; LDL-C: low-density lipoprotein cholesterol; HDL-C: high-density lipoprotein cholesterol.

**Table S13. Meta-analysis of rs3211956 with EOCAD.**

| **Groups or subgroups** | ***P*_H_** | **OR (95% CI)** | ***P*_OR_** |  | **Groups or subgroups** | ***P*_H_** | **OR (95% CI)** | ***P*_OR_** |
| --- | --- | --- | --- | --- | --- | --- | --- | --- |
| ***Overall results*** | | | |  | ***Recalculated results that eliminated heterogeneity*** | | | |
| Allelic model (G vs. T) |  |  |  |  | Allelic model (G vs. T) |  |  |  |
| All | 0.09 | 0.81 (0.59-1.11)  04 | 0.19 |  | All | 0.92 | 0.68 (0.53-0.88)  04 | <0.01 |
| Studies in HWE | 0.09 | 0.81 (0.59-1.11)  04 | 0.19 |  | Studies in HWE | 0.92 | 0.68 (0.53-0.88)  04 | <0.01 |
| Asian | 0.92 | 0.68 (0.53-0.88) | <0.01 |  | Asian | 0.92 | 0.68 (0.53-0.88)  04 | <0.01 |
| Additive model (GG vs. TT) |  |  |  |  | Additive model (GG vs. TT) |  |  |  |
| All | 0.41 | 0.66 (0.38-1.15)  04 | 0.14 |  | All | 0.29 | 0.59 (0.31-1.13)  04 | 0.11 |
| Studies in HWE | 0.41 | 0.66 (0.38-1.15)  04 | 0.14 |  | Studies in HWE | 0.29 | 0.59 (0.31-1.13)  04 | 0.11 |
| Asian | 0.29 | 0.59 (0.31-1.13)  04 | 0.11 |  | Asian | 0.29 | 0.59 (0.31-1.13)  04 | 0.11 |
| Dominant model (TG+GG vs. TT) |  |  |  |  | Dominant model (TG+GG vs. TT) |  |  |  |
| All | 0.02 | 0.73 (0.44-1.21)  04 | 0.23 |  | All | 0.54 | 0.59 (0.43-0.82)  04 | <0.01 |
| Studies in HWE | 0.02 | 0.73 (0.44-1.21)  04 | 0.23 |  | Studies in HWE | 0.54 | 0.59 (0.43-0.82)  04 | <0.01 |
| Asian | 0.54 | 0.59 (0.43-0.82)  04 | <0.01 |  | Asian | 0.54 | 0.59 (0.43-0.82)  04 | <0.01 |
| Recessive model (TG+TT vs. GG) |  |  |  |  | Recessive model (TG+TT vs. GG) |  |  |  |
| All | 0.12 | 0.84 (0.32-2.22)  04 | 0.72 |  | All | 0.12 | 0.84 (0.32-2.22)  04 | 0.72 |
| Studies in HWE | 0.12 | 0.84 (0.32-2.22)  04 | 0.72 |  | Studies in HWE | 0.12 | 0.84 (0.32-2.22)  04 | 0.72 |
| Asian | 0.12 | 0.84 (0.32-2.22)  04 | 0.72 |  | Asian | 0.12 | 0.84 (0.32-2.22)  04 | 0.72 |

EOCAD: early-onset coronary artery disease; SMD: standardized mean difference; 95% CI: 95% confidence interval; *P*_H_: *P*_Heterogeneity_; HWE: Hardy-Weinberg equilibrium; TG: triglycerides; TC: total cholesterol; LDL-C: low-density lipoprotein cholesterol; HDL-C: high-density lipoprotein cholesterol.

**
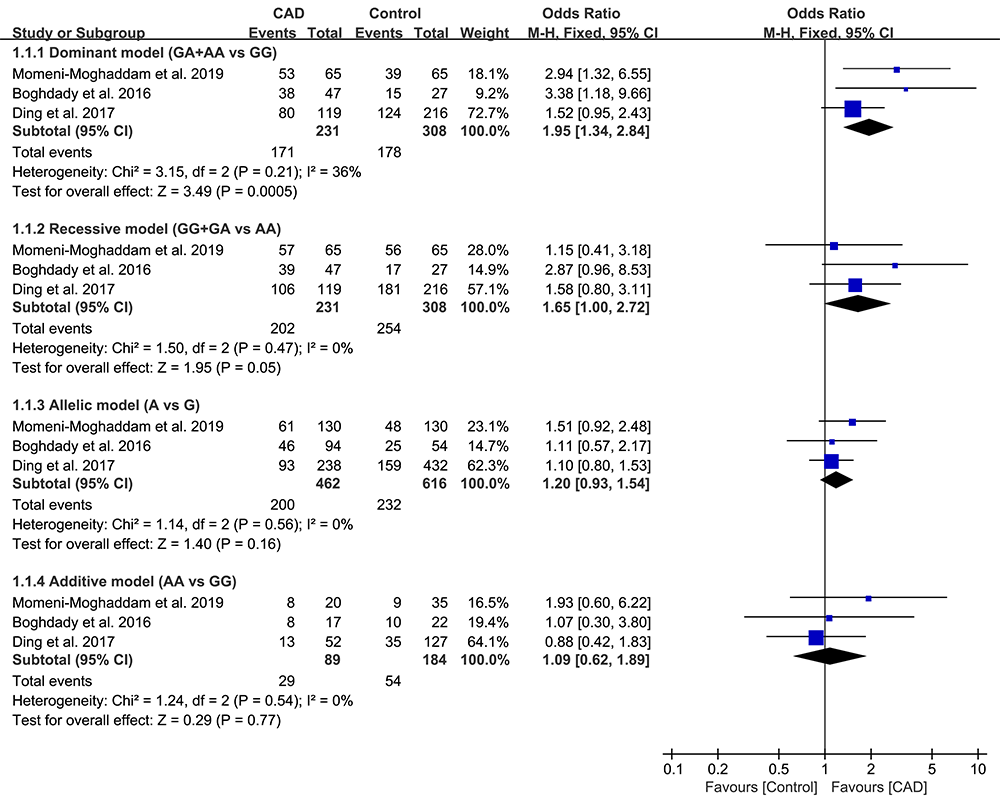
**

**Figure S1.** Forest plot for rs1761667 with EOCAD.


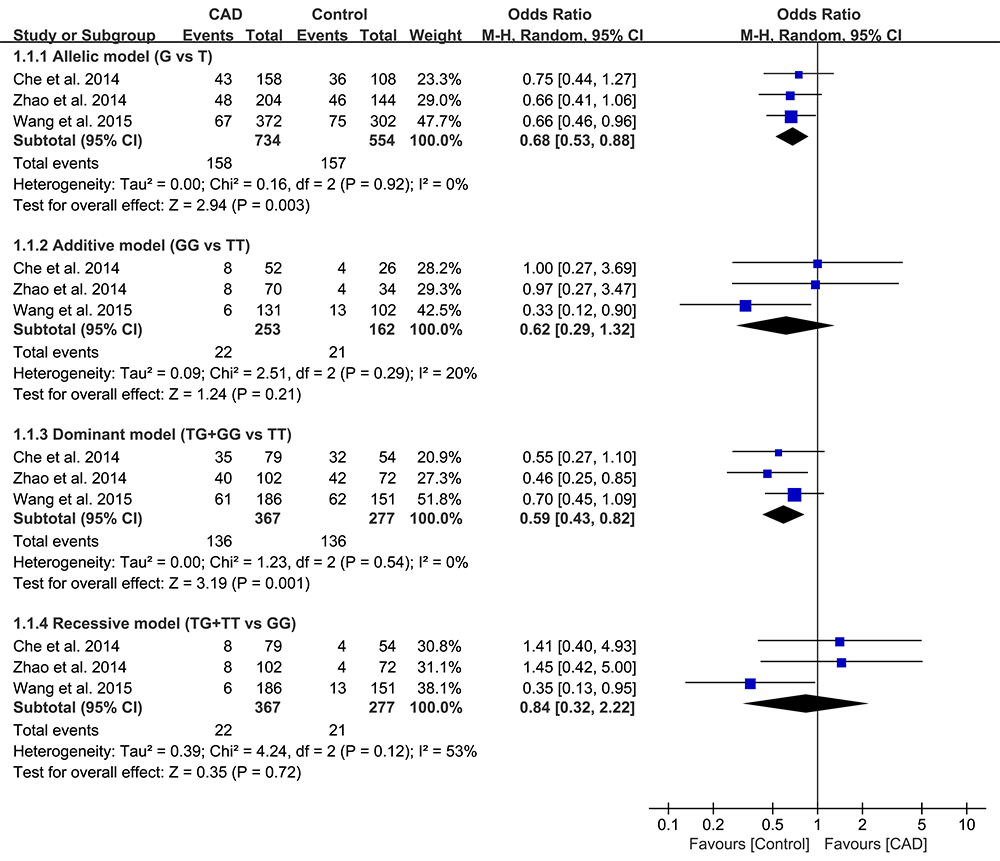


**Figure S2.** Forest plot for rs3211956 with EOCAD.

**
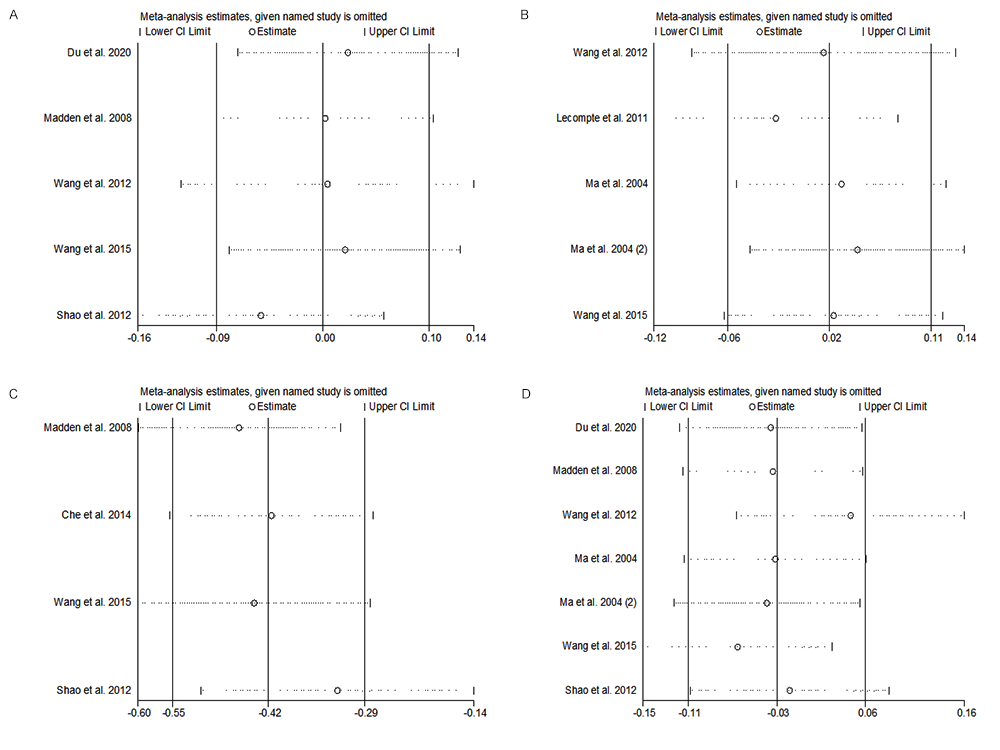
**

**Figure S3.** Sensitivity analysis for rs1049673 with lipid levels. Open circle is SMD, parallel lines represent 95% CI (A: rs1049673 with TG levels; B: rs1049673 with TC levels; C: rs1049673 with LDL-C levels; D: rs1049673 with HDL-C levels).

**
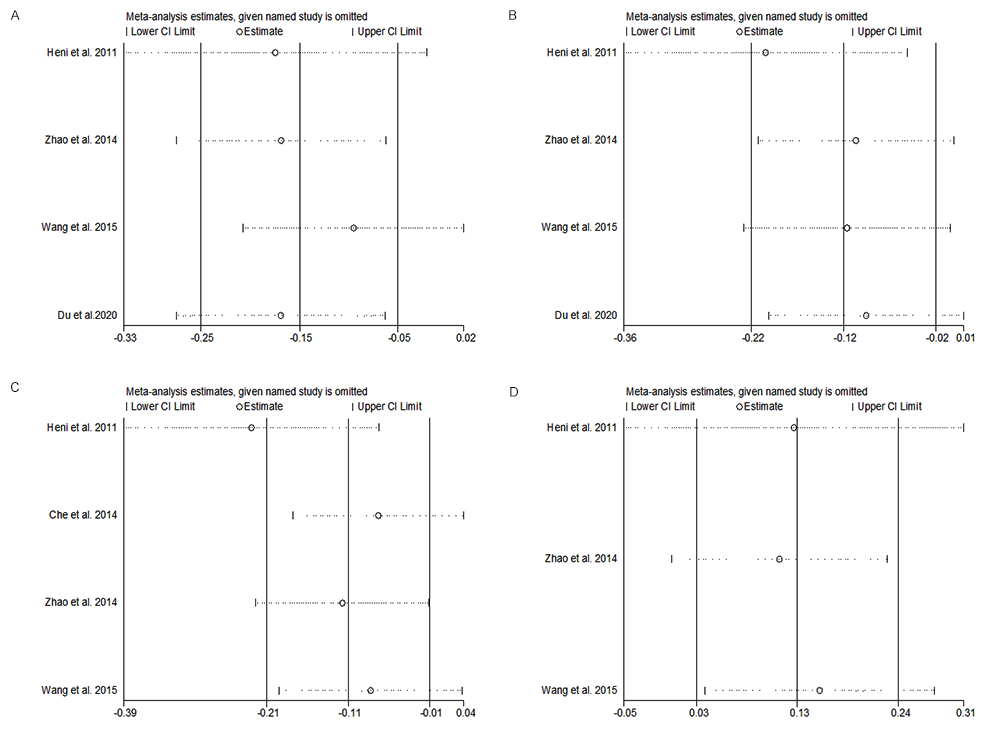
**

**Figure S4.** Sensitivity analysis for rs3211956 with lipid levels. Open circle is SMD, parallel lines represent 95% CI (A: rs3211956 with TG levels; B: rs3211956 with TC levels; C: rs3211956 with LDL-C levels; D: rs3211956 with HDL-C levels).


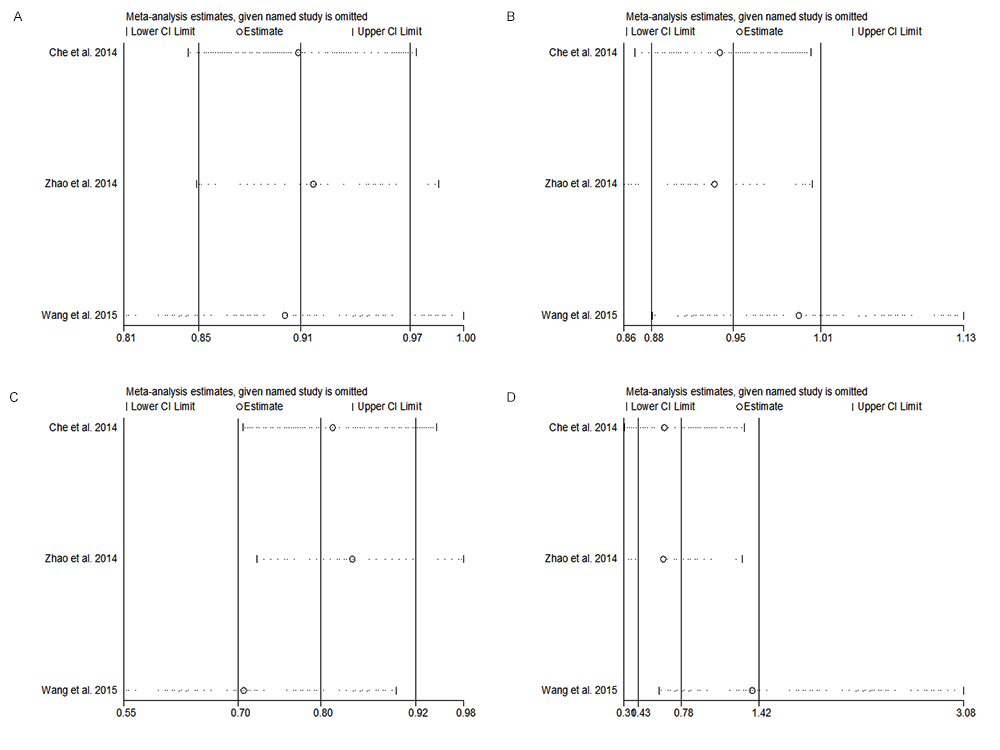


**Figure S5.** Sensitivity analysis for rs3211956 with EOCAD. Open circle is OR, parallel lines represent 95% CI [A: allelic model (G vs. T); B: additive model (GG vs. TT); C: dominant model (TG+GG vs. TT ); D: recessive model (TG+TT vs. GG)].

**
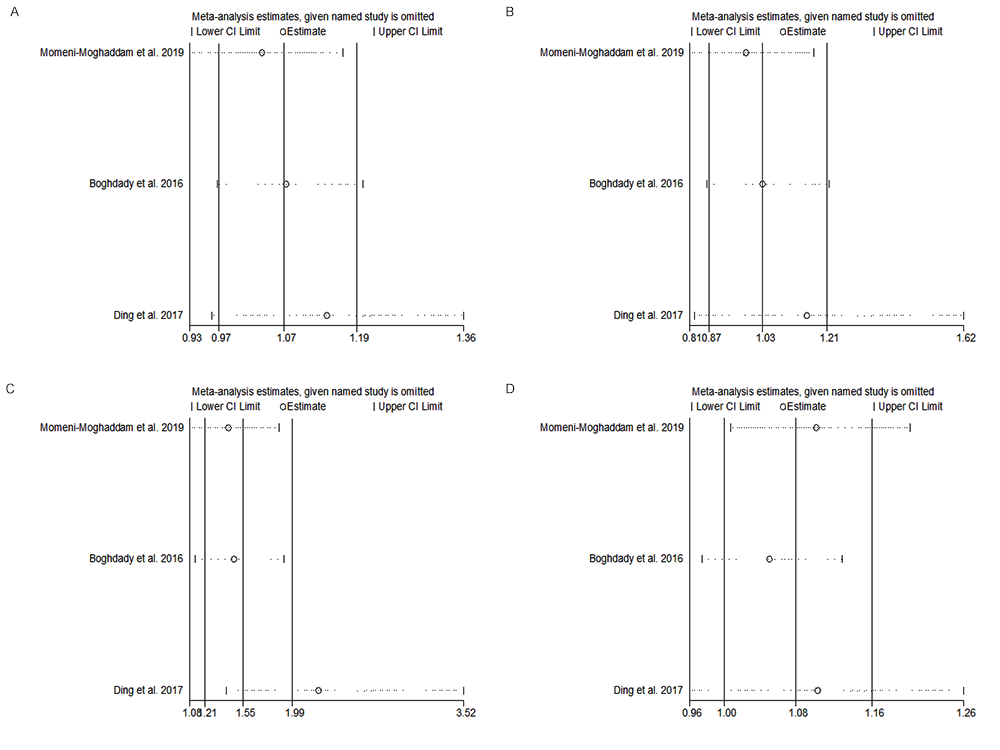
**

**Figure S6.** Sensitivity analysis for rs1761667 with EOCAD. Open circle is OR, parallel lines represent 95% CI [A: allelic model (A vs. G); B: additive model (AA vs. GG); C: dominant model (GA+AA vs. GG ); D: recessive model (GA+GG vs. AA)].


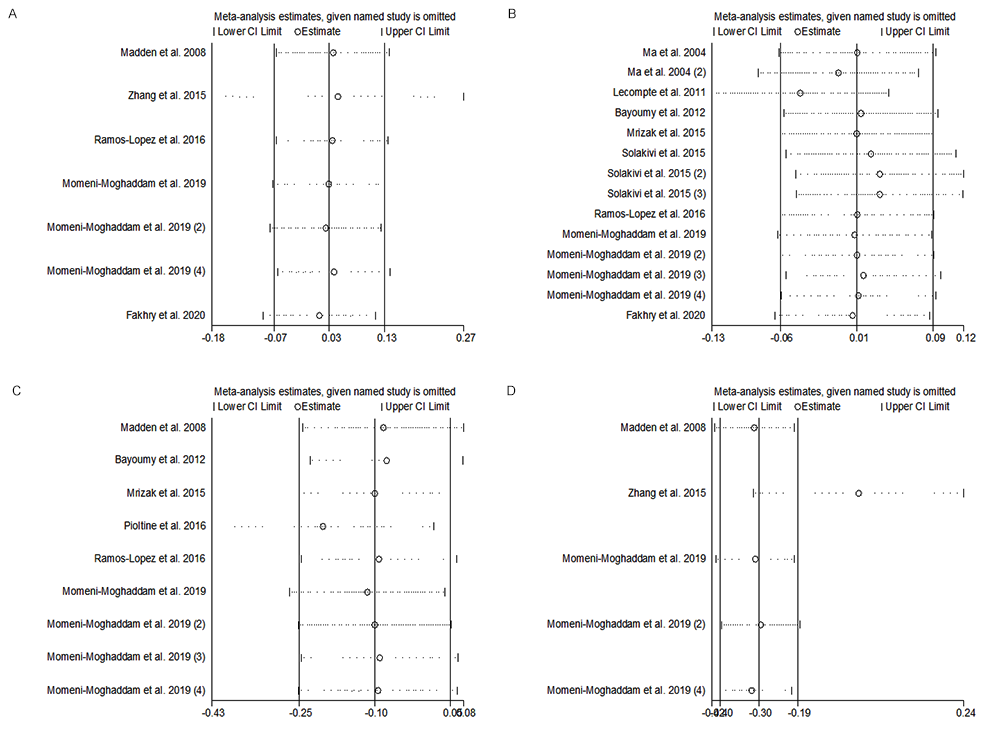


**Figure S7.** Sensitivity analysis for rs1761667 with lipid levels. Open circle is SMD, parallel lines represent 95% CI (A: rs1761667 with TG levels; B: rs1761667 with TC levels; C: rs1761667 with LDL-C levels; D: rs1761667 with HDL-C levels).


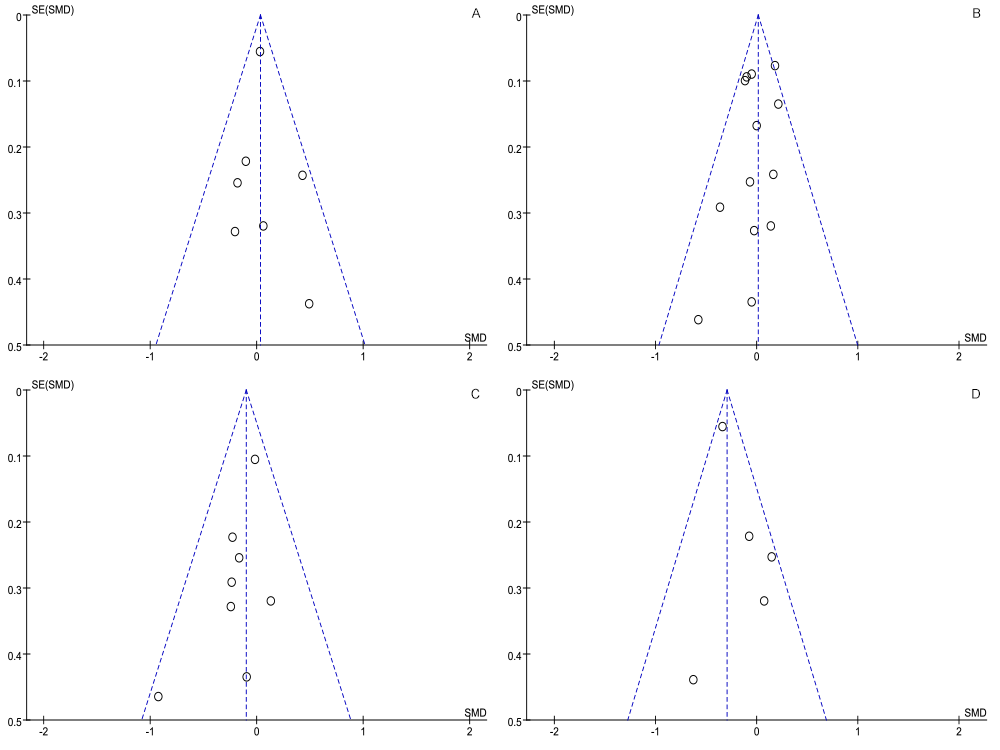


**Figure S8.** Begg’s funnel plot for rs1761667 with lipid levels. Each small circle represents a separate study, the diverging lines represent 95% CI and the central line is SMD. [A: rs1761667 with TG levels (*P* = 0.78); B: rs1761667 with TC levels (*P* = 0.43); C: rs1761667 with LDL-C levels (*P* = 0.10); D: rs1761667 with HDL-C levels (*P* = 0.27)]

**
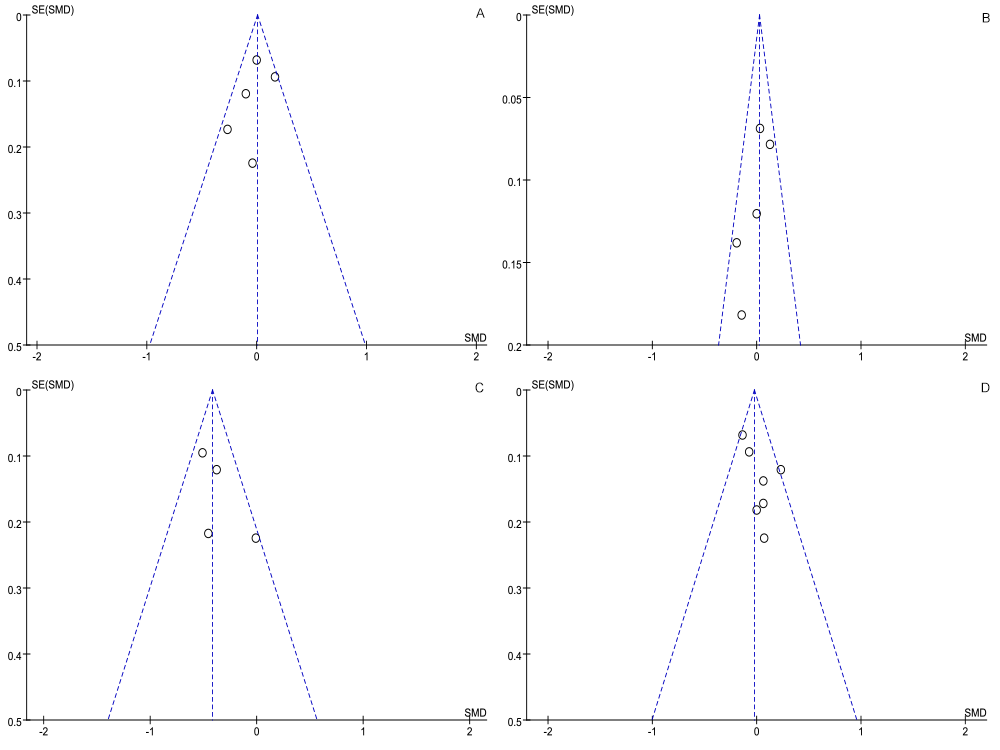
**

**Figure S9.** Begg’s funnel plot for rs1049673 with lipid levels. Each small circle represents a separate study, the diverging lines represent 95% CI and the central line is SMD. [A: rs1049673 with TG levels (*P* = 0.43); B: rs1049673 with TC levels

(*P* = 0.13); C: rs1049673 with LDL-C levels (*P* = 0.29); D: rs1049673 with HDL-C levels (*P* = 0.12)]


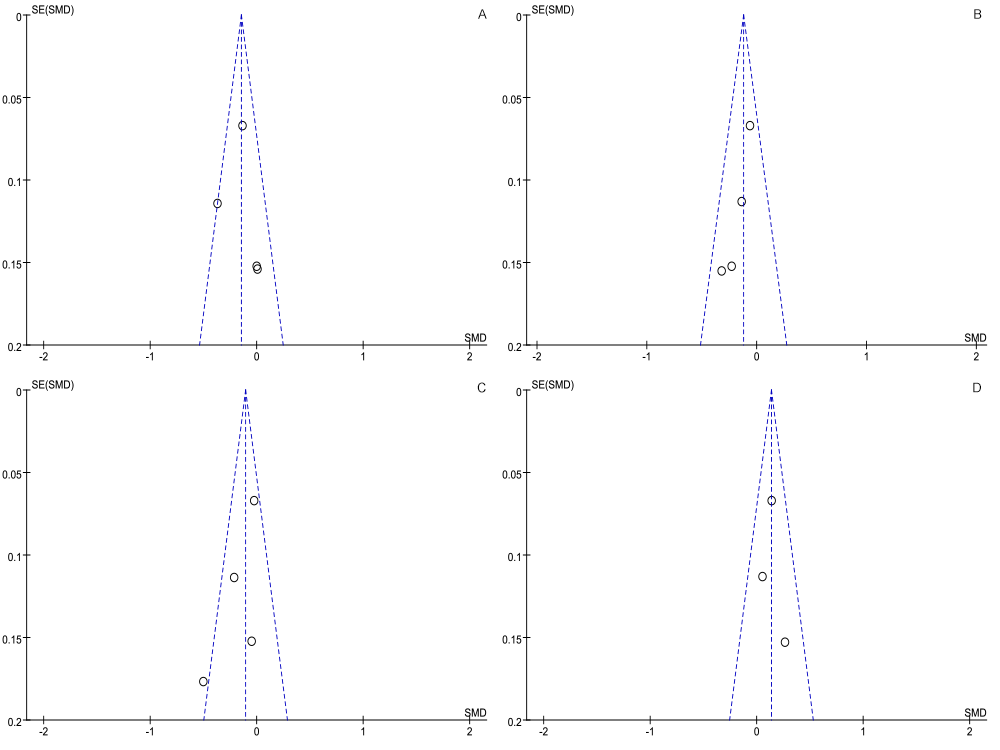


**Figure S10.** Begg’s funnel plot for rs3211956 with lipid levels. Each small circle represents a separate study, the diverging lines represent 95% CI and the central line is SMD. [A: rs3211956 with TG levels (*P* = 0.81); B: rs3211956 with TC levels

(*P* = 0.19); C: rs3211956 with LDL-C levels (*P* = 0.24); D: rs3211956 with HDL-C levels (*P* = 0.79)]

**
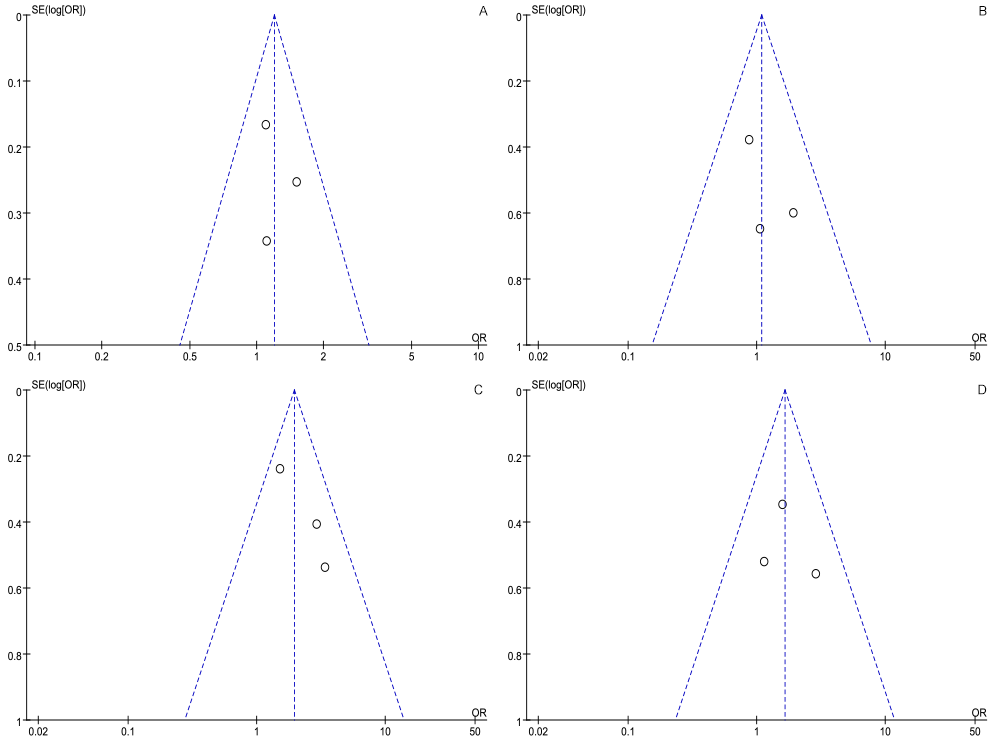
**

**Figure S11.** Begg’s funnel plot for rs1761667 with EOCAD. Each small circle represents a separate study, the diverging lines represent 95% CI and the central line is SMD. [A: allelic model (*P* = 0.75); B: additive model (*P* = 0.54); C: dominant model (*P* = 0.11); D: recessive model (*P* = 0.72)].

**
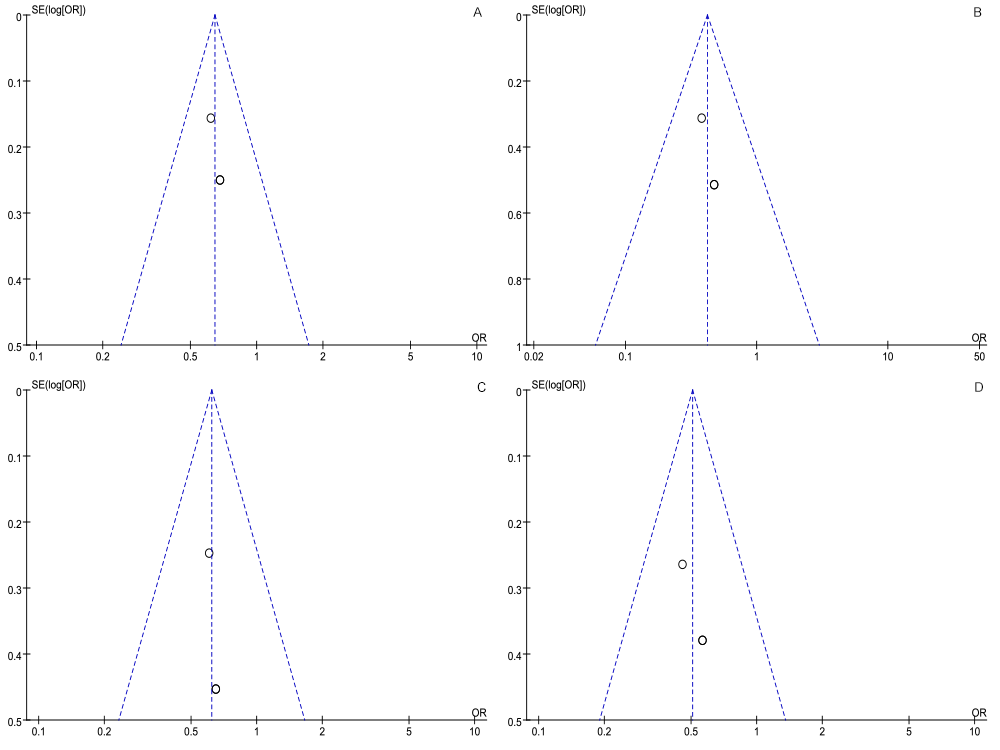
**

**Figure S12.** Begg’s funnel plot for rs1049673 with EOCAD. Each small circle represents a separate study, the diverging lines represent 95% CI and the central line is SMD. [A: allelic model (*P* = 0.30); B: additive model (*P* = 0.60); C: dominant model (*P* = 0.30); D: recessive model (*P* = 0.60)].

**
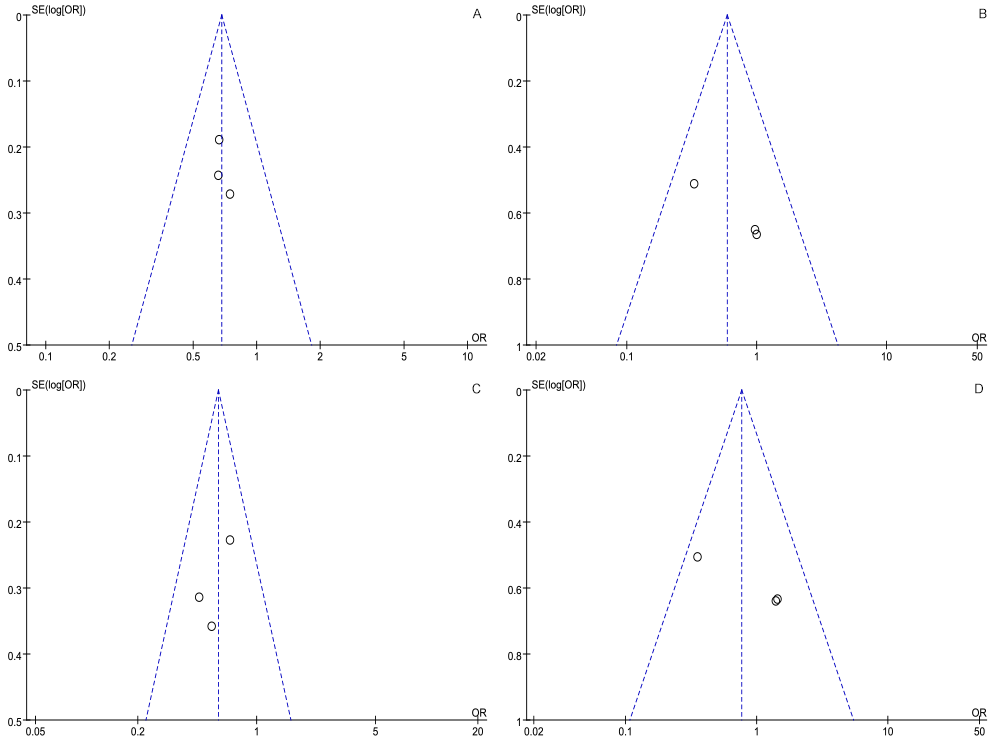
**

**Figure S13.** Begg’s funnel plot for rs3211956 with EOCAD. Each small circle represents a separate study, the diverging lines represent 95% CI and the central line is SMD. [A: allelic model (*P* = 0.53); B: additive model (*P* = 0.12); C: dominant model (*P* = 0.36); D: recessive model (*P* = 0.60)].
